# Major Components of Energy Drinks (Caffeine, Taurine, and Guarana) Exert Cytotoxic Effects on Human Neuronal SH-SY5Y Cells by Decreasing Reactive Oxygen Species Production

**DOI:** 10.1155/2013/791795

**Published:** 2013-05-22

**Authors:** Fares Zeidán-Chuliá, Daniel Pens Gelain, Eduardo Antônio Kolling, José Luiz Rybarczyk-Filho, Priscilla Ambrosi, Silvia Resende Terra, André Simões Pires, João Batista Teixeira da Rocha, Guilherme Antônio Behr, José Cláudio Fonseca Moreira

**Affiliations:** ^1^Department of Biochemistry, Center of Oxidative Stress Research, Institute of Basic Health Sciences, Federal University of Rio Grande do Sul (UFRGS), 90035-003 Porto Alegre, RS, Brazil; ^2^Departamento de Física e Biofísica, Instituto de Biociências de Botucatu, Universidade Estadual Paulista (UNESP), 18618-970 Botucatu, SP, Brazil; ^3^Departamento de Química, Centro de Ciências Naturais e Exatas (CCNE), Universidade Federal de Santa Maria (UFSM), 97105-900 Santa Maria, RS, Brazil; ^4^Department of Psychiatry and Behavioral Neurosciences, McMaster University, Hamilton, ON, Canada L8P 3B6

## Abstract

*Scope*. To elucidate the morphological and biochemical *in vitro* effects exerted by caffeine, taurine, and guarana, alone or in combination, since they are major components in energy drinks (EDs). *Methods and Results*. On human neuronal SH-SY5Y cells, caffeine (0.125–2 mg/mL), taurine (1–16 mg/mL), and guarana (3.125–50 mg/mL) showed concentration-dependent nonenzymatic antioxidant potential, decreased the basal levels of free radical generation, and reduced both superoxide dismutase (SOD) and catalase (CAT) activities, especially when combined together. However, guarana-treated cells developed signs of neurite degeneration in the form of swellings at various segments in a beaded or pearl chain-like appearance and fragmentation of such neurites at concentrations ranging from 12.5 to 50 mg/mL. Swellings, but not neuritic fragmentation, were detected when cells were treated with 0.5 mg/mL (or higher doses) of caffeine, concentrations that are present in EDs. Cells treated with guarana also showed qualitative signs of apoptosis, including membrane blebbing, cell shrinkage, and cleaved caspase-3 positivity. Flow cytometric analysis confirmed that cells treated with 12.5–50 mg/mL of guarana and its combinations with caffeine and/or taurine underwent apoptosis. *Conclusion*. Excessive removal of intracellular reactive oxygen species, to nonphysiological levels (or “antioxidative stress”), could be a cause of *in vitro* toxicity induced by these drugs.

## 1. Introduction

**Figure 1:**
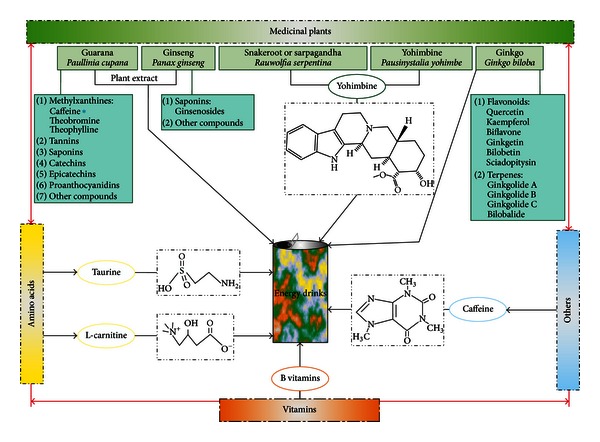
Schematic representations illustrating the different components found in EDs. EDs are highly caffeinated beverages which may contain herbal supplements, alkaloids from different plants, vitamins, or even amino acids. In some cases, the addition of herbal supplements (e.g., guarana) can increase the caffeine content of these beverages (*). For our *in vitro* study, we selected caffeine, taurine, and guarana as major components of EDs.

Worldwide consumption of energy drinks (EDs) is exponentially increasing due to their stimulant effect on the central nervous system and body and the purpose of enhancing both cognitive and physical performances. EDs are highly caffeinated and may contain herbal supplements (e.g., guarana), alkaloids found in different plants (e.g., yohimbine), vitamins (e.g., B vitamins), and taurine (Figure 1) [1, 2]. For instance, one single can of one of the most consumed brand of EDs (Red Bull, 250 mL) contains 80 mg of caffeine (or 0.32 mg/mL) and 1000 mg of taurine (or 4 mg/mL) as major components. Other brands commonly add guarana in the composition (e.g., Monster Energy, Rockstar, and 180). Furthermore, it has been reported that the number of energy drinks consumed in one session reaches an average of 5 units (cans) in one single night [3].

 The concentration of caffeine in EDs is significantly higher than those found in cola drinks, and such amounts are known to cause a variety of adverse health effects [4]. Also, the use of some herbal supplements in these beverages, like guarana, increases the amount of caffeine and other active methylxanthines that multiply any potential toxicity. 

The widespread consumption of EDs is becoming especially popular among people below the age of 25 and adolescents [1, 2]. Nonetheless, only a limited number of studies have tried to evaluate the short- and long-term effects of ED consumption. Most of them examined the cognitive effects derived from the use of these drinks or some of their major components (e.g., caffeine or taurine), alone or in coadministration with alcohol [5, 6]. Some other reports are highlighting the possible link between the use of highly caffeinated beverages and the increased propensity for addiction to other substances like alcohol or even new-onset seizures in adults [7, 8]. 

 Different countries have already started to regulate the sale of highly caffeinated EDs to prevent potential health problems. Denmark and France banned the sale of some brands; in Norway, some EDs are only available in pharmacies; in general, the European Union demands the labeling of highly caffeinated drinks [1, 2]. Major concerns are arising with the impact these beverages may have on children and adolescents' developing brains [9]. Therefore, in the present study, we used a well-known neurotoxicity cell culture model (human neuronal SH-SY5Y cells) [10] in order to elucidate the potential toxicity (synergistic or not) of caffeine, taurine, and guarana, three components that are commonly present in EDs at high concentrations. 

## 2. Materials and Methods

### 2.1. Drugs

Guarana (*Paullinia cupana Mart.*) powder was obtained from Lifar Ltd. (Porto Alegre, RS, Brazil). According to the manufacturer, 50 mg of guarana powder (obtained from grounding of dried seeds) contains approximately 2 mg of caffeine. Both caffeine and taurine were purchased from Sigma Chemical Co. (St. Louis, MO, USA).

### 2.2. Cell Culture and Treatments

Human neuroblastoma-derived SH-SY5Y cells were purchased from Rio de Janeiro Cell Bank ((BCRJ) Rio de Janeiro, Brazil). The cells were cultured in a 1 : 1 mixture of Ham's F12 and Dulbecco Modified Eagle Medium (DMEM) supplemented with 10% heat-inactivated fetal bovine serum (FBS), 2 mM of glutamine, 0.28 mg/mL of gentamicin, and 250 *μ*g of amphotericin B in a 5% CO_2_ humidified incubator at 37°C. Cells were subcultured until they reached 80–90% of confluence and then trypsinized. 24 hours after trypsinization, approximately half of volume of old cell culture medium was replaced with fresh 10% FBS-DMEM: F12 medium. All treatments were performed when cells reached a confluence of approximately 75% followed by 2-hour incubation in complete absence of FBS; except for one experiment (long-term effects on cell number after 24 hours of treatment with ED components or their combinations, Figure 11) where no previous FBS deprivation was performed. For each assay, guarana (Gua), caffeine (Caf), and taurine (Tau) at concentrations of 50 mg/mL, 2 mg/mL, and 16 mg/mL, respectively, were dissolved in either DMEM: F12 medium (without FBS) or 10% FBS-DMEM: F12 medium (Figure 11), and serial dilutions were obtained from this stock solution. 

### 2.3. Phase Contrast Microscopy (PCM) Analysis

PCM micrographs were taken by using an inverted microscope (Nikon Eclipse TE300) connected to a digital camera.

### 2.4. Scanning Electron Microscopy (SEM) Analysis

SH-SY5Y cells were exposed to each drug treatment for 4 hours, fixed in 2.5% glutaraldehyde, treated with osmium tetroxide in distilled water, dehydrated with sequential washes in 30%, 50%, 70%, 90%, and 100% acetone, and then dried in critical point dryer by using a Balzers CPD-030 instrument before coating with gold in a Balzers SCD 050 sputter coater. Samples were visualized in a JEOL-JSM-6060 scanning electron microscope.

### 2.5. Confocal Immunofluorescence Microscopy (CM) Analysis

Cells were washed with phosphate-buffered saline (PBS) and fixed on chamber slides with 4% paraformaldehyde (PFA) in 4°C for 15 min. Samples were sequentially treated with 0.15% Triton X-100 (for permeabilization) for 10 min and 10% serum (to avoid nonspecific binding) for 30 min. Cells were immunostained with specific antibodies after 4 hours of treatment with guarana, caffeine, and taurine. Primary antibodies were utilized as follows: rabbit anticleaved caspase-3 (Cell Signaling Technology, 9664, 1 : 1000), mouse anti-*β*-III tubulin (Novex, 480011, 1 : 1000), and fluorescent-labeling Alexa Fluor 488 goat anti-mouse (Molecular Probes, A11001, 1 : 1000), fluorescent-labeling Alexa Fluor 488 goat anti-rabbit (Molecular Probes, A11008, 1 : 1000). For nuclear staining, Prolong Gold Antifade Reagent with DAPI (Molecular Probes, P36931) was used. As a marker for cellular viability, together with *β*-III tubulin (antineutron-specific antibody), cells were co-immunostained with propidium iodide (PI) (BD Pharmingen). Images were taken with an Olympus FluoView 1000 confocal microscope and subsequently analyzed by using Olympus FluoView FV1000 Software, ver. 3.0. Thresholds discriminating between signal and background were selected by utilizing cells that were only stained with secondary antibodies in order to discriminate any false positive result.

### 2.6. Total Reactive Antioxidant Potential (TRAP) and Total Antioxidant Reactivity (TAR)

TRAP and TAR were measured and calculated as previously described [11]. Briefly, TRAP represents the nonenzymatic antioxidant capacity of the cells once they are treated (in this study, with guarana, caffeine, and taurine). This is determined by measuring the luminol chemiluminescence intensity of emission induced by thermolysis of  2,2′-azobis (2-amidinopropane) hydrochloride (AAPH) as free radical source. The system was left to stabilize for 2 hours. Then, samples were added and the readings monitored for 2 hours. Results were transformed into a percentile rank, and the area under the curve (AUC) was calculated by utilizing the GraphPad software (San Diego, CA, USA), as previously described. The smaller the AUC is (in comparison to the system), the higher the total reactive antioxidant potential of the sample is. TAR represents the total antioxidant properties of all antioxidants. This parameter is closely related to the quality of the antioxidants within the sample. In our study, TAR was calculated as the ratio of light in the absence of samples (*I*_o_)/light intensity right after sample addition (*I*). The higher these values are, the higher the total antioxidant reactivity of the sample is.

### 2.7. Intracellular Reactive Oxygen Species Production (DCFH-DA Assay)

Intracellular reactive species production was measured by the DCFH-DA assay, as previously described [11]. This technique is based on the capability of DCFH to be oxidized to highly fluorescent dichlorofluorescein (DCF) in the presence of reactive oxygen species (ROS). This can be used as an index to measure the level of free radical production in cells. Briefly, SH-SY5Y cells were seeded in 96-well plates. 100 *μ*M DCFH-DA was then dissolved in medium containing 1% FBS and added to each well. Cells were incubated for 2 hours in order to allow cellular incorporation. Thereafter, this medium was discarded, and cells were treated with guarana (12 mg/mL), caffeine (0.5 mg/mL), taurine (4 mg/mL), and their different combinations. H_2_O_2_ 1 mM was used as positive control for DCF fluorescence. At this endpoint, DCF fluorescence was read at 37°C in a fluorescence plate reader (Spectra Max M2, Molecular Devices, USA) with an emission wavelength of 535 nm and an excitation wavelength of 485 nm. The results were expressed as percentage of DCF fluorescence in relation to untreated control.

### 2.8. Cellular Antioxidant Enzyme Activities

In order to determine the cellular antioxidant enzyme activities, cells were collected, resuspended, and homogenized in 50 mM PBS at pH 7.4. The resulting cellular suspension was centrifuged at 3000 ×g for 10 min, and the supernatant was collected. Cellular superoxide dismutase (SOD) activity was assessed by quantifying the inhibition of superoxide-dependent adrenaline autooxidation in a spectrophotometer at 480 nm, as previously described [12]. Results were expressed as units of SOD/mg protein. Cellular catalase (CAT) activity was determined by measuring the rate of decrease in H_2_O_2_ absorbance in a spectrophotometer at 240 nm [12]. CAT activity was expressed as units of CAT/mg protein. Cellular glutathione peroxidase (GPx) activity was assessed by measuring the rate of NADPH oxidation in a spectrophotometer at 340 nm, as previously described [12]. GPx activity was expressed as units (nmol NADPH oxidized/min)/mg protein. Cellular glutathione S-transferase (GST) activity was determined in a reaction mixture with 1 mM 1-chloro-2,4-dinitrobenzene (CDNB) and 1 mM glutathione as substrates, as previously described [13]. GST activity was expressed as units of GST/mg protein.

### 2.9. Measurement of Protein Thiol Content

This assay is utilized for analyzing oxidative alterations in proteins by measuring the level of reduced thiol content (SH) in the samples [14]. Briefly, each aliquot was diluted in 10 mM 5,5′-dithiobis-2-nitrobenzoic acid in ethanol. After 60 minutes of incubation at 25°C, the reaction gives rise to an intense yellow color which is then read in a spectrophotometer at 412 nm. The results were expressed as *μ*mol SH/mg protein.

### 2.10. Measurement of Thiobarbituric Acid Reactive Species (TBARS)

Formation of TBARS is widely accepted as an index of lipid peroxidation, as previously described [12]. Briefly, the samples were mixed with 1 mL of trichloroacetic acid (TCA) 10% and 1 mL of thiobarbituric acid (TBA) 0.67% and then heated in a boiling water bath for 15 min. 1,1,3,3-tetramethoxypropane (TMP) was used as a TBARS standard. TBARS were determined by the absorbance at 535 nm and were expressed as nmol TMP/mg protein.

### 2.11. Flow Cytometric (FC) Analyses

Analysis of granularity versus size: the side (SSC-H) and forward (FSC-H) scatter parameters distinguish the cells from one another based on internal complexity and size, respectively. To examine these morphological characteristics, SH-SY5Y cells were cultured in 6-well plates, preincubated for 2 hours in FBS-free culture medium, and then treated with guarana, caffeine, and taurine for 4 hours in DMEM: F12 medium (without FBS). Then, cells were analyzed by flow cytometry (BD FACSCalibur flow cytometer, BD Biosciences); data were presented as dot plots (SSC-H/granularity plotted against FSC-H/size) and finally analyzed by FlowJo (cytometric data analysis and presentation software). 

Analysis of cell death (necrosis versus apoptosis): SH-SY5Y cells were cultured in 6-well plates, preincubated for 2 hours in FBS-free culture medium, and then treated with guarana, caffeine, taurine, and their combinations for 4 hours in DMEM: F12 medium (without FBS). The FITC Annexin V apoptosis detection kit (BD Pharmingen) was then utilized in order to distinguish apoptotic from necrotic events. Briefly, cells were washed with PBS and resuspended in binding buffer. Thereafter, Annexin V and PI were added and left for incubation (15 minutes) at room temperature (25°C) in the complete absence of light. Then, cell death was measured by using flow cytometry (BD FACSCalibur flow cytometer, BD Biosciences). Data are presented as dot plots (PI plotted against Annexin V) and analyzed by FlowJo (cytometric data analysis and presentation software). Ten thousand cells were analyzed per sample, and data were also reported as the percentage of late necrotic cells (Q1: quadrant 1, upper left), early necrotic/late apoptotic cells (Q2: quadrant 2, upper right), early apoptotic (Q3: quadrant 3, lower right), and living cells (Q4: quadrant 4, lower left). 

Analysis of drug-induced long-term effects on cell number: for examining the *in vitro* long-term effects of guarana, caffeine, taurine, and their combinations, SH-SY5Y cells were cultured in 6-well plates and treated in the presence of 10% FBS-DMEM: F12 medium for 24 hours. Then, after collecting the cells, the total cell count was determined by using the flow cytometer (BD FACSCalibur flow cytometer, BD Biosciences).

### 2.12. *In Silico* Network Development and Determination of Centralities to Predict the Relevance of Genes/Proteins in the Overall Architecture of the MEDRI Model

The *in silico* network model of interactions between ED components within redox/nitric oxide (NO) [11] and apoptotic pathways (KEGG pathway database; http://www.genome.jp/kegg/pathway.html) was developed by interconnecting 16 compounds (12 ED components, hydrogen peroxide, hydroxyl radicals, molecular oxygen, and nitric oxide) and 144 proteins (87 apoptosis-related and 57 redox/NO-related proteins) based on their possible interactions through either “activation,” “inhibition,” “catalysis,” “binding,” or “reaction.” The network was generated by using the database resource search tool STRING 9.0 (http://string-db.org/) for the retrieval of interacting genes [15] with “Databases” and “Experiments” as input options and a confidence score of 0.400 (medium confidence). STRING provides a public database with information about direct and indirect functional protein-protein associations/interactions. Proteins were identified by the HUGO Gene Symbol [16] and Ensembl protein ID [17]. Then, small molecule-small molecule and small molecule-protein interactions were found by using STITCH 3.0 (http://stitch.embl.de/) [18], with “Databases” and “Experiments” as input options and a confidence score of 0.400 (medium confidence). The links between two different nodes (protein-protein, compound-compound, and protein-compound) provided by STRING 9.0 and STITCH 3.0 are saved in data files to be handled in the Medusa interface [19].

The complete list with gene symbols, compound names, and IDs (Ensembl protein IDs and Compound IDs, resp.,) is additionally provided (see Supporting Information Tables  S1, S2, and S3 available online at http://dx.doi.org/10.1155/2013/791795). 

 For elucidating the topological network properties, Cytoscape (http://www.cytoscape.org/), an open source platform for complex network analysis and visualization, was used [20]. Numeric values concerning the properties of each node are also provided (Supporting Information Table S4). Finally, to visualize the topological network properties of each node within MEDRI network model, numeric values were projected in a 2D color representation by utilizing ViaComplex software (http://lief.if.ufrgs.br/pub/biosoftwares/viacomplex/) [21].

### 2.13. Protein Quantification

The protein content of each sample was measured by Lowry method [22].

### 2.14. Statistical Analysis

Results were expressed as the mean ± SEM of three independent experiments (*n* = 3). Data were analyzed by a one-way analysis of variance (ANOVA) followed by Dunnett's multiple comparison test. Differences were considered to be significant when *P < 0.05, **P < 0.01, or ***P < 0.001.

## 3. Results

### 3.1. Distinct Morphological Alterations on Human Neuronal Cells Triggered by Guarana, Taurine, and Caffeine: Signs of Apoptosis and Neurite Degeneration

Firstly, we characterized the morphological events induced by separate treatments of human neuronal cells (SH-SY5Y) with increasing concentrations of either guarana (3.125, 12.5, and 50 mg/mL), caffeine (0.125, 0.5, and 2 mg/mL), or taurine (1, 4, and 16 mg/mL). For each drug, we selected a range of concentrations based on (I) the content of these three compounds that one can find in different brands of EDs, available for human consumption in Brazil, and according to the information provided in the label (i.e., caffeine and taurine concentrations in one popular brand are 0.32 and 4 mg/mL, resp.); (II) that already* in vitro* (Chinese hamster ovary cells (CHO cells)), doses of aqueous extracts from guarana powder (*Paullinia cupana)* ranging from 10 to 40 mg/mL exhibited significant differences in cytotoxicity [23]; (III) that Bydlowski and colleagues (1988) [24], with the goal of finding new therapeutic uses of guarana, found that concentrations of 100 mg/mL of guarana decreased thromboxane synthesis in blood platelets as well as reversed and inhibited platelet aggregation and postulated that guarana could be useful in preventing cardiovascular disease; (IV) that any potential effects induced by the studied concentrations of guarana and caffeine, in this and further experiments of the current study, could be compared since 50 mg of guarana (Lifar Ltd.; Porto Alegre, RS, Brazil) contains approximately 2 mg of caffeine, according to the manufacturer (as indicated in Section 2). 

 Thus, to perform such characterization, we decided to use PCM analysis after short-term drug treatments (2 hours), and, in order to look for further morphological features (changes in growth pattern, cell morphology, size, granularity, or any marker of interest), we consequently analyzed the samples after longer treatments (medium-term treatment of 4 hours) by using SEM, FC (side scatter versus forward scatter), and CM. 

**Figure 2:**
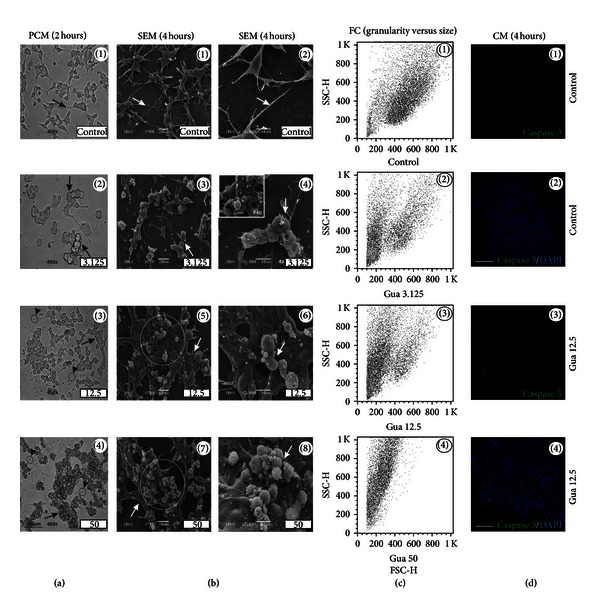
Guarana-induced morphological alterations in human neuronal SH-SY5Y cells. (a) Phase contrast microscopy (PCM) of SH-SY5Y cells after treatments with different concentrations of guarana (3.125, 12.5, and 50 mg/mL) for 2 hours in FBS-free culture medium. Scale bar represents 30 *μ*m. (b) Scanning electron microscopy (SEM) of SH-SY5Y cells after treatments with different concentrations of guarana (3.125, 12.5, and 50 mg/mL) for 4 hours in FBS-free culture medium. Scale bars represent 20 or 10 *μ*m (for ×700 or ×2000, resp.). (c) Analysis of cellular complexity or granularity (side scatter, SSC-H) and size (forward scatter, FSC-H) by flow cytometry (FC), after treatment with different concentrations of guarana (3.125, 12.5, and 50 mg/mL) for 4 hours in FBS-free culture medium. (d) Confocal immunofluorescence microscopy (CM) of cleaved caspase-3 expression (green fluorescence) and DAPI (nuclei) on SH-SY5Y cells, after treatment with 12.5 mg/mL of guarana for 4 hours in FBS-free culture medium. Scale bars represent 10 *μ*m.

Under the cell culture conditions utilized for the expansion of our *in vitro* model (see Section 2), aggregates of SH-SY5Y cells are rare, and they usually grow in monolayers with “flattened-like” phenotype (Figures 2(a)(1) and 2(b)(1)) with some cytoplasmic projections (Figure 2(b)(2)). However, in the presence of low concentrations of guarana (3.125 mg/mL), contiguous tracts of cells were often observed within the culture (Figures 2(a)(2) and 2(b)(3)). Also, the membrane of some cells looked disrupted in a higher or lower grade (Figure 2(b)(4)). In some cells, single or very few membrane blebs were detected (Figure 2(b)(4b)) at such concentration. On the other hand, at higher concentrations of guarana (12.5 and 50 mg/mL), the majority of the cells started to aggregate into clusters and shrunk (arrows, Figures 2(a)(3) and 2(a)(4)). Membrane integrity did not look compromised, but both number and shape of the blebs were considerably increased (Figures 2(b)(5)–2(b)(8)). Moreover, the presence of neuritic abnormalities after treatment with 12.5 or 50 mg/mL of guarana was remarkable. Treated cells (12.5 and 50 mg/mL) displayed neuritic swellings and shortened neurites (by either retraction or lack of growth) (arrowheads in Figures 2(a)(3) and 2(a)(4)). Since the predominant signatures of apoptosis, in addition to membrane blebbing, include the loss of cell volume and higher granularity, we performed FC analysis (SSC-H versus FSC-H) after treating the cells for 4  hours with increasing concentrations of guarana. Qualitatively, increasing concentrations of guarana correlated with a decrease in cell size (FSC-H) and increased granularity (SSC-H) (Figure 2(c)). Thus, in order to search for further evidence of apoptotic process, we immunostained the cells with a specific anticleaved caspase-3 antibody together with DAPI (nuclear staining) and then analyzed the cells by confocal microscopy. Cleaved caspase-3 expression was detected in cells treated with 12.5 mg/mL of guarana for 4 hours (Figure 2(d)(3)). 

**Figure 3:**
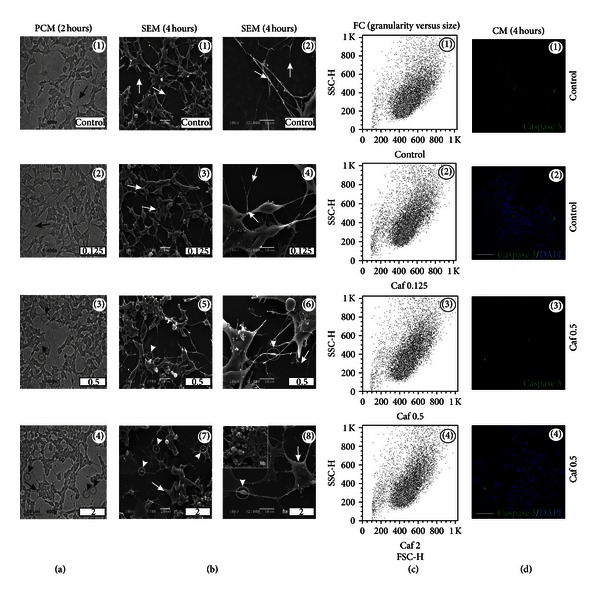
Caffeine-induced morphological alterations in human neuronal SH-SY5Y cells. (a) Phase contrast microscopy (PCM) of SH-SY5Y cells after treatments with different concentrations of caffeine (0.125, 0.5, and 2 mg/mL) for 2 hours in FBS-free culture medium. Scale bar represents 30 *μ*m. (b) Scanning electron microscopy (SEM) of SH-SY5Y cells after treatments with different concentrations of caffeine (0.125, 0.5, and 2 mg/mL) for 4 hours in FBS-free culture medium. Scale bars represent 20 or 10 *μ*m (for ×700 or ×2000, resp.). (c) Analysis of cellular complexity or granularity (side scatter, SSC-H) and size (forward scatter, FSC-H) by flow cytometry (FC), after treatments with different concentrations of caffeine (0.125, 0.5, and 2 mg/mL) for 4 hours in FBS-free culture medium. (d) Confocal immunofluorescence microscopy (CM) of cleaved caspase-3 expression (green fluorescence) and DAPI (nuclei) on SH-SY5Y cells, after treatment with 0.5 mg/mL of caffeine for 4 hours in FBS-free culture medium. Scale bars represent 10 *μ*m.

Treatments with 0.125 mg/mL of caffeine, for either 2 or 4 hours, did not show any clear morphological alteration in SH-SY5Y cells (black and white arrows in Figures 3(a)(1), 3(a)(2), and 3(b)(1)–3(b)(4)). On the other hand, cells exposed to 0.5 and 2 mg/mL of caffeine (2 or 4 hours) exhibited neuritic swellings (black and white arrowheads; Figures 3(a)(3), 3(a)(4), and 3(b)(5)–3(b)(8a)). When cells were treated with 2 mg/mL of caffeine, one could also find few cells with mild signs of membrane blebbing (Figure 3(b)(8b)). However, no significant changes in size, granularity, or cleaved caspase-3 expression were observed when compared to control cells (Figures 3(c) and 3(d)).

**Figure 4:**
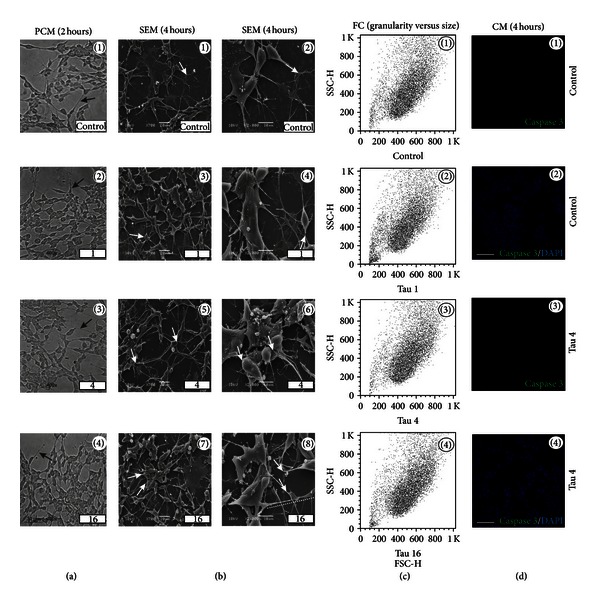
Taurine-induced morphological alterations in human neuronal SH-SY5Y cells. (a) Phase contrast microscopy (PCM) of SH-SY5Y cells after treatments with different concentrations of taurine (1, 4, and 16 mg/mL) for 2 hours in FBS-free culture medium. Scale bar represents 30 *μ*m. (b) Scanning electron microscopy (SEM) of SH-SY5Y cells after treatments with different concentrations of taurine (1, 4, and 16 mg/mL) for 4 hours in FBS-free culture medium. Scale bars represent 20 or 10 *μ*m (for ×700 or ×2000, resp.). (c) Analysis of cellular complexity or granularity (side scatter, SSC-H) and size (forward scatter, FSC-H) by flow cytometry (FC), after treatment with different concentrations of taurine (1, 4, and 16 mg/mL) for 4 hours in FBS-free culture medium. (d) Confocal immunofluorescence microscopy (CM) of cleaved caspase-3 expression (green fluorescence) and DAPI (nuclei) on SH-SY5Y cells, after treatment with 4 mg/mL of taurine for 4 hours in FBS-free culture medium. Scale bars represent 10 *μ*m.

In contrast to guarana and caffeine, taurine-treated cells exhibited healthy neuritic processes with long cytoplasmic projections (Figures 4(a) and 4(b)), without changes in cell shape or complexity (Figure 4(c)). Neither blebs (Figure 4(b)) nor cleaved caspase-3 positive cells were found (Figure 4(d)) after taurine treatment. 

**Figure 5:**
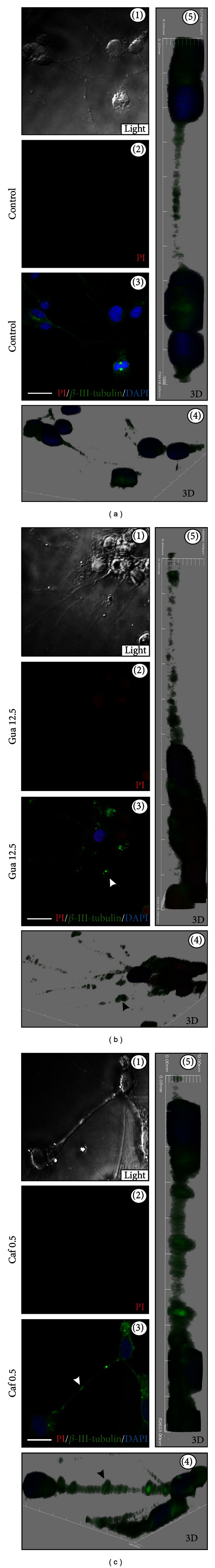
Qualitative analysis of drug-induced neuritic morphology. Confocal immunofluorescence microscopy (CM) of *β*-III-tubulin expression (green fluorescence), propidium iodide (PI) (red fluorescence), and DAPI (nuclei) on human neuronal SH-SY5Y cells (a), after treatment with 12.5 mg/mL of guarana (b) and 0.5 mg/mL of caffeine (c) for 4 hours in FBS-free culture medium. White and black arrow heads (in 2D and 3D representations) mark the presence of neuritic swelling after guarana (12.5 mg/mL) and caffeine (0.5 mg/mL) treatments. Scale bars represent 10 *μ*m.

**Figure 6:**
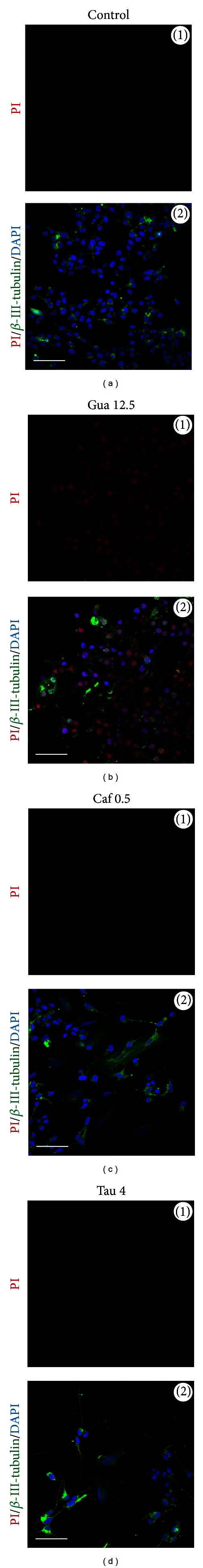
Qualitative analysis of drug-induced changes on cellular viability. Confocal immunofluorescence microscopy (CM) of propidium iodide (PI) (red fluorescence), *β*-III-tubulin expression (green fluorescence), and DAPI (nuclei) on human neuronal SH-SY5Y cells (a), after treatment with 12.5 mg/mL of guarana (b), 0.5 mg/mL of caffeine (c), and 4 mg/mL of taurine (d) for 4 hours in FBS-free culture medium. Scale bars represent 10 *μ*m.

Even though guarana-(12.5 and 50 mg/mL) and caffeine-(0.5 and 2 mg/mL) treated cells showed neuritic swellings, CM analysis with *β*-III tubulin antibody (neuron-specific marker) and PI (marker for cellular viability) highlighted some differences. Formation of pearl chain-like structures along the neurite was present in both treatments (Figures 5(b)(3)–5(b)(5) and 5(c)(3)–5(c)(5)). However, guarana-treated neurites looked shrunken and fragmented (Figures 5(b)(4) and 5(b)(5)) when compared to caffeine-treated cells (Figures 5(c)(4) and 5(c)(5)). In contrast to caffeine and taurine, the viability of guarana-treated cells was compromised as shown in the figure with PI positivity (Figure 6). 

### 3.2. Antioxidant Potential and Intracellular ROS Scavenging in SH-SY5Y Cells after Treatments with Guarana, Caffeine, Taurine, or Their Combinations

**Figure 7:**
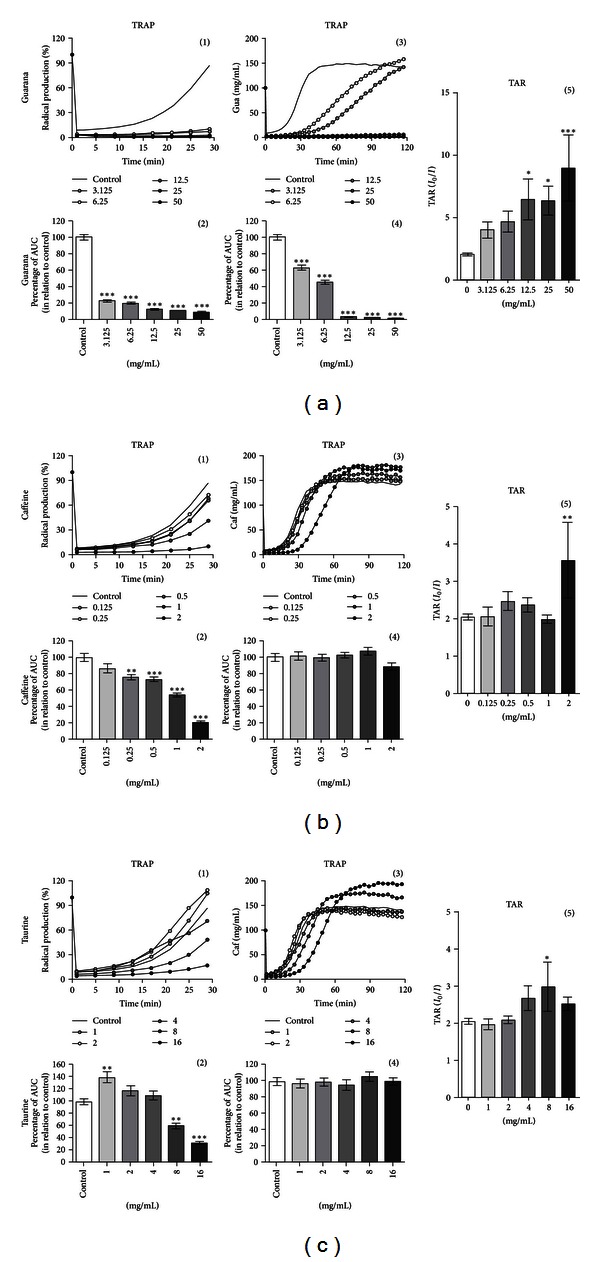
Total reactive antioxidant potential (TRAP) and total antioxidant reactivity (TAR) on human neuronal SH-SY5Y cells exposed to major components of EDs. TRAP and TAR measurements on guarana-treated cells (2 hours) at concentrations of 3.125, 6.25, 12.5, 25, and 50 mg/mL (a), caffeine-treated cells (2 hours) at concentrations of 0.125, 0.25, 0.5, 1, and 2 mg/mL (b), and taurine-treated cells (2 hours) at concentrations of 1, 2, 4, 8, and 16 mg/mL (c). TRAP is shown as a representative graphic of the area under the curve (AUC) for 2 hours, and TAR is calculated as the ratio of light in the absence of samples (*I*_0_)/light intensity right after sample addition (*I*). Values are expressed as mean ± SEM of three independent experiments (*n* = 3). Statistical difference compared to control was determined by one-way ANOVA followed by Dunnett's multiple comparison test (*P < 0.05; **P < 0.01; ***P < 0.001).

Many of the components present in EDs (e.g., taurine, guarana, or carnitine) are known to exert antioxidant activity in different biological systems [25–27]. Thus, we evaluated the nonenzymatic antioxidant potential *in vitro*, exerted after 2 hours of treatment with different concentrations of guarana (3.125–50 mg/mL), caffeine (0.125–2 mg/mL), and taurine (1–16 mg/mL) by using the TRAP and TAR assays (Figure 7). By utilizing the TRAP assay (which is more related to the amount of the antioxidant), an increase of nonenzymatic antioxidant potential was observed in guarana-treated cells at all tested concentrations when compared to control cells (***P < 0.001), as shown in the graph by the decrease in the AUC (Figures 7(a)(2) and 7(a)(4)). These effects were maintained during approximately 120 minutes of analysis. Moreover, TAR analysis (more related to scavenger capacity or quality of the antioxidant) also showed significant differences between control and guarana-treated cells, when cells were exposed to either 12.5 (*P < 0.05), 25 (*P < 0.05), or 50 mg/mL (***P < 0.001) (Figure 7(a)(5)). 

At concentrations of 0.25 (**P < 0.01), 0.5 (***P < 0.001), 1 (***P < 0.001), and 2 mg/mL (***P < 0.001) of caffeine, an increase of nonenzymatic antioxidant potential was also detected in the cells (Figure 7(b)(2)), which lasted for approximately 30 minutes. In this case, a significant difference in the total antioxidant reactivity of caffeine-treated cells was observed at the highest concentration of 2 mg/mL (**P < 0.01) (Figure 7(b)(5)).

Interestingly, 2 hours of treatment with the lowest concentration of taurine (1 mg/mL) seems to exert prooxidant effects (**P < 0.01) on SH-SY5Y cells (Figure 7(c)(2)). In contrast, higher concentrations of the drug (8 and 16 mg/mL) increased the nonenzymatic antioxidant potential (**P < 0.01 and ***P < 0.001, resp.). Such effect was maintained for approximately 30 minutes of analysis. Thereafter, no significant changes were detected (Figure 7(c)(4)). TAR assay showed that exclusively 8 mg/mL of taurine was able to increase the total antioxidant reactivity (*P < 0.05) on the cells (Figure 7(c)(5)).

**Figure 8:**
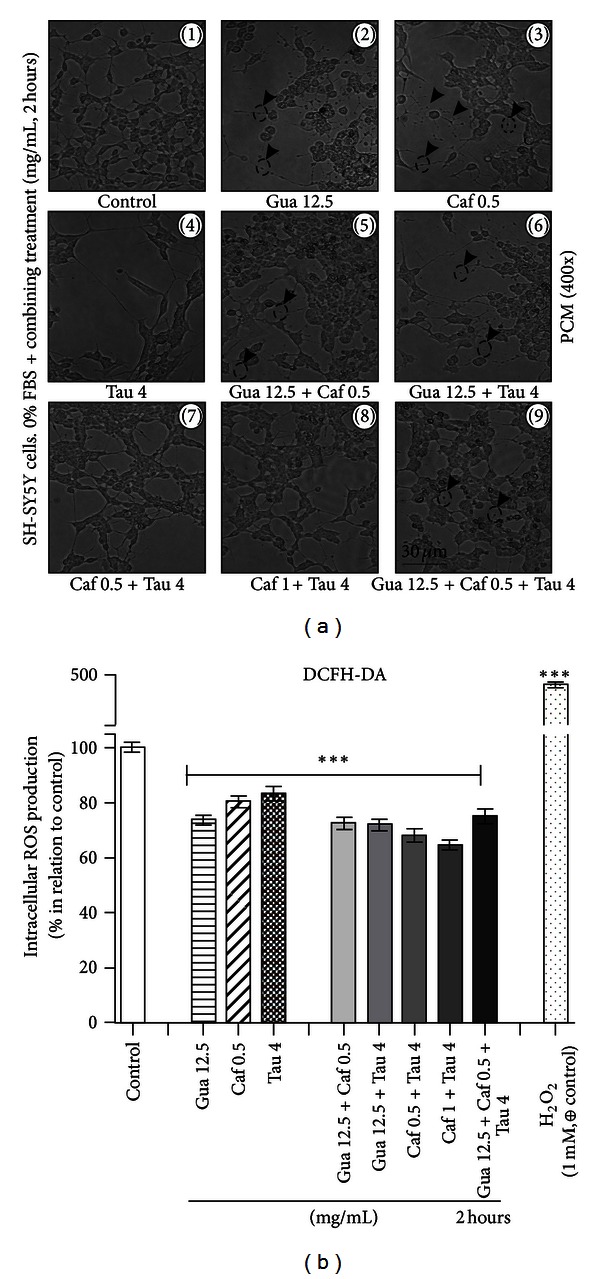
Morphological alterations of human neuronal SH-SY5Y cells treated with drug combinations (guarana, caffeine, and taurine) and effects on the intracellular production of reactive oxygen species (ROS). (a) Phase contrast microscopy (PCM) of human neuronal SH-SY5Y cells after treatments with guarana (12.5 mg/mL), caffeine (0.5 mg/mL), taurine (4 mg/mL), and their combinations for 2 hours in FBS-free culture medium. Scale bar represents 30 *μ*m. (b) Intracellular ROS production was examined by incubating the cells for 2 hours with 100 *μ*M of DCFH-DA dissolved in 1% FBS-containing culture medium. Then, medium was discarded, and human neuronal SH-SY5Y cells were treated with H_2_O_2_ (1 mM; positive control for intracellular ROS production), guarana (12.5 mg/mL), caffeine (0.5 mg/mL), taurine (4 mg/mL), and their combinations for 2 hours. Finally, DCF fluorescence was read (endpoint; 2 hours) at 37°C in a fluorescence plate reader (Spectra Max M2, Molecular Devices, USA) with an emission wavelength set at 535 nm and an excitation wavelength set at 485 nm. Values are expressed as mean ± SEM of three independent experiments (*n* = 3). Statistical difference compared to control was determined by one-way ANOVA followed by Dunnett's multiple comparison test (*P < 0.05; **P < 0.01; ***P < 0.001).

 In order to determine the potential effect of these drugs on the production of intracellular ROS, we used the DCFH-DA assay. Cells were treated for 2 hours with 12.5 mg/mL of guarana, 0.5 mg/mL of caffeine, 4 mg/mL of taurine, and their different combinations (12.5 mg/mL of guarana + 0.5 mg/mL of caffeine; 12.5 mg/mL of guarana + 4 mg/mL of taurine; 0.5 mg/mL of caffeine + 4 mg/mL of taurine; 1 mg/mL of caffeine + 4 mg/mL of taurine; 12.5 mg/mL of guarana + 0.5 mg/mL of caffeine + 4 mg/mL of taurine). All the tested treatments significantly decreased (***P < 0.001) the basal levels of free radical generation (endpoint; 2 hours) (Figure 8(b)). PCM analysis confirmed the presence of neuritic swellings (arrowheads in Figure 8(a)) when cells were exposed to 12.5 mg/mL of guarana, 0.5 mg/mL of caffeine, and also combinations of guarana (12.5 mg/mL) with caffeine (0.5 mg/mL) and/or taurine (4 mg/mL).

### 3.3. Significant Decrease of SOD and CAT Activities in SH-SY5Y Cells Exposed to Guarana and Its Combinations with Caffeine and/or Taurine

**Figure 9:**
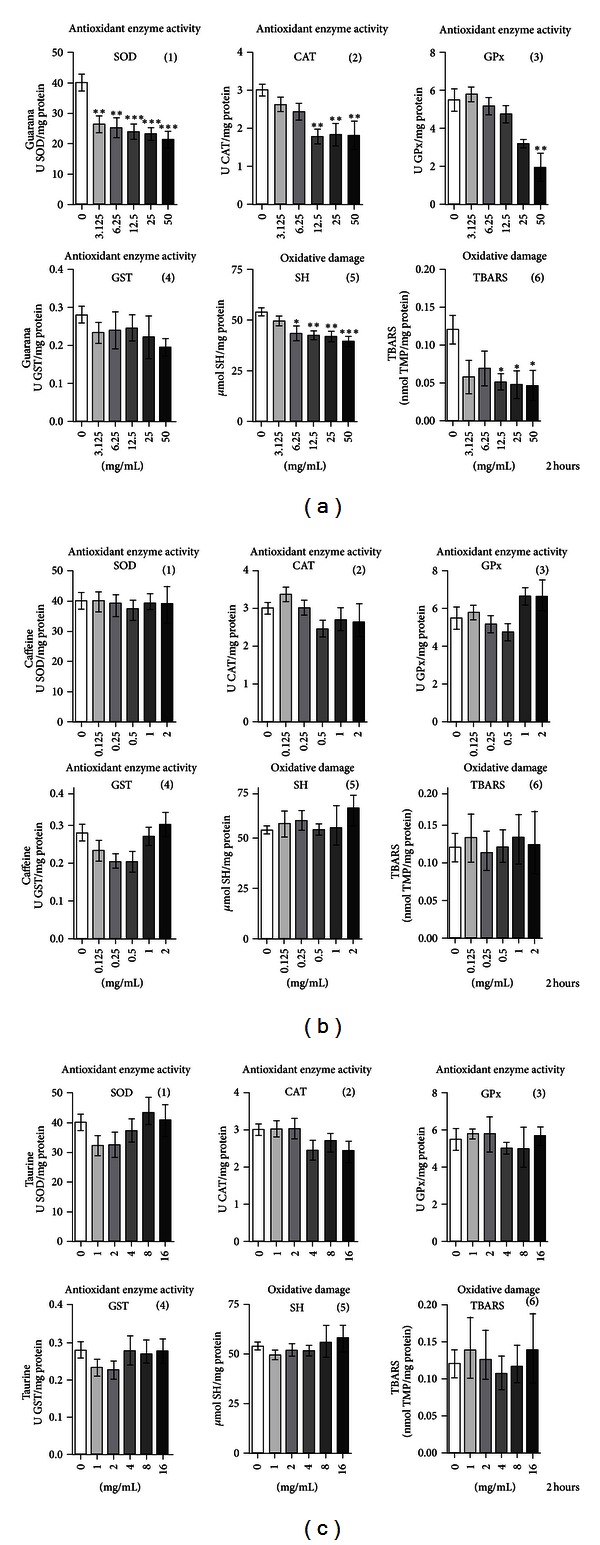
Antioxidant enzyme activity and oxidative damage parameters on human neuronal SH-SY5Y cells exposed to major components of EDs. Enzymatic activity (SOD, CAT, GPx, and GST), SH, and TBARS measurements on guarana-treated cells (2 hours) at concentrations of 3.125, 6.25, 12.5, 25, and 50 mg/mL (a), caffeine-treated cells (2 hours) at concentrations of 0.125, 0.25, 0.5, 1, and 2 mg/mL (b), and taurine-treated cells (2 hours) at concentrations of  1, 2, 4, 8, and 16 mg/mL (c). Values are expressed as mean ± SEM of three independent experiments (*n* = 3). Statistical difference compared to control was determined by one-way ANOVA followed by Dunnett's multiple comparison test (*P < 0.05; **P < 0.01; ***P < 0.001).

**Figure 10:**
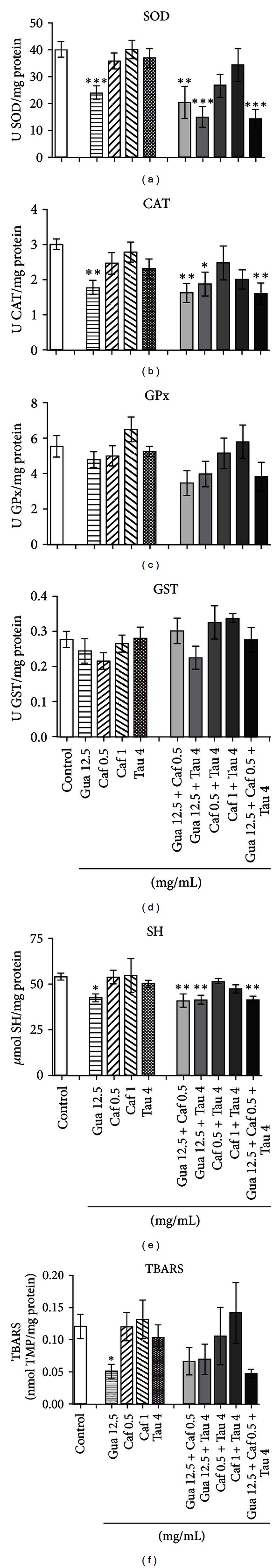
Antioxidant enzyme activity and oxidative damage parameters on human neuronal SH-SY5Y cells treated for 2 hours with drug combinations (guarana, caffeine, and taurine). Measurements for SOD (a), CAT (b), GPx (c), GST (d), SH (e), and TBARS (f) at different drug concentrations (mg/mL) are shown. Values are expressed as mean ± SEM of three independent experiments (*n* = 3). Statistical difference compared to control was determined by one-way ANOVA followed by Dunnett's multiple comparison test (*P < 0.05; **P < 0.01; ***P < 0.001).

For screening any potential variability in the antioxidant enzyme activity of SH-SY5Y cells after 2 hours of drug treatment with guarana, caffeine, taurine, and their combinations, the cellular activities of SOD, CAT, GPx, and GST were analyzed. Guarana-treated cells showed an exponential decrease in SOD activity (Figure 9(a)(1)) at concentrations ranging from 3.125 to 50 mg/mL (3.125 and 6.25 mg/mL, **P < 0.01; 12.5, 25, and 50 mg/mL, ***P < 0.001). Statistical analysis did not reveal significant differences for caffeine- or taurine-treated cells (Figures 9(b)(1) and 9(c)(1)). Combinations of caffeine and/or taurine with 12.5 mg/mL of guarana (12.5 mg/mL of guarana + 0.5 mg/mL of caffeine; 12.5 mg/mL of guarana + 4 mg/mL of taurine; 12.5 mg/mL of guarana + 0.5 mg/mL of caffeine + 4 mg/mL of taurine) also induced a significant decrease in SOD activity *in vitro* (Figure 10(a)). Similar results were obtained for CAT, with significant decrease in the enzymatic activity at concentrations of guarana ranging from 12.5 to 50 mg/mL (**P < 0.01) (Figure 9(a)(2)), but no significant differences were detected for both caffeine- and taurine-treated cells (Figures 9(b)(2) and 9(c)(2)). Again, combinations of caffeine and/or taurine with 12.5 mg/mL of guarana (12.5 mg/mL of guarana + 0.5 mg/mL of caffeine; 12.5 mg/mL of guarana + 4 mg/mL of taurine; 12.5 mg/mL of guarana + 0.5 mg/mL of caffeine + 4 mg/mL of taurine) also decreased CAT activity in the cells (Figure 10(b)).

Analyses of GPx and GST activities under the same conditions did not show significant variations, except for a decrease in cellular GPx activity at 50 mg/mL of guarana treatment (Figures 9(a)(3), 10(c), and 10(d)). Statistical analysis of SOD/CAT + GPx ratio by ANOVA followed by Dunnett's multiple comparison test did not reveal any imbalance (data not shown).

### 3.4. Guarana Protects SH-SY5Y Cells against Oxidative Alterations of Proteins and Lipids

It is well known that protein thiols can maintain the cellular redox status by their cysteine content [28]. Lipid peroxidation, on the other hand, is one of the main causes of free radical-mediated damage to cellular membranes [28]. Therefore, we investigated the *in vitro* effects of these drugs and their combinations against oxidative alterations in both proteins and lipids. Our results showed that only guarana treatment protected against lipid peroxidation (12.5–50 mg/mL; *P < 0.05) (Figure 9(a)(6)). Though ANOVA followed by Dunnett's multiple comparison test did not show significant differences in lipid peroxidation when cells were treated with the combination of guarana (12.5 mg/mL), caffeine (0.5 mg/mL), and taurine (4 mg/mL) (Figure 10(f)), the Student's *t*-test revealed, on the other hand, a significant difference when compared to control cells (*P* = 0.0162; statistical significance when *P* < 0.05). Additionally, concentrations of guarana ranging from 6.25 to 50 mg/mL and all combinations where this drug was present decreased (*P < 0.05 and **P < 0.01) the total reduced thiol content (Figures 9(a)(5) and 10(e)), demonstrating protective effects against oxidative alterations of proteins. 

### 3.5. Increasing Concentrations of Guarana and Its Combinations with Caffeine and/or Taurine Decrease the Number of Viable SH-SY5Y Cells by Elevating the Percentage of Late Apoptotic Cells

**Figure 11:**
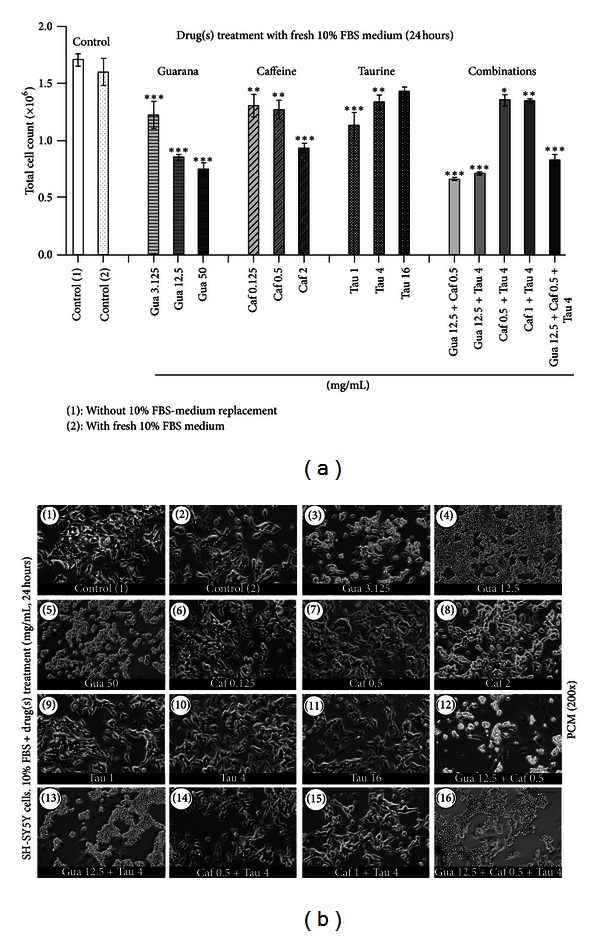
Analysis of drug-induced long-term effects on cell number. (a) Flow cytometric analysis (FC) of the total number of human neuronal SH-SY5Y cells after treatments with guarana (3.125, 12.5, and 50 mg/mL), caffeine (0.125, 0.5, and 2 mg/mL), taurine (1, 4, and 16 mg/mL), and their combinations for 24 hours in 10% FBS-containing culture medium. Values are expressed as mean ± SEM of three independent experiments (*n* = 3). Statistical difference compared to control (1) was determined by one-way ANOVA followed by Dunnett's multiple comparison test (*P < 0.05; **P < 0.01; ***P < 0.001). (b) Phase contrast microscopy (PCM) of human neuronal SH-SY5Y cells after treatments with different concentrations of guarana (3.125, 12.5, and 50 mg/mL), caffeine (0.125, 0.5, and 2 mg/mL), taurine (1, 4, and 16 mg/mL), and their combinations for 24 hours in 10% FBS-containing culture medium. Scale bar represents 30 *μ*m.

For elucidating the drug-induced effects on the cellular number, cells were seeded in 6-well plates. 48 hours later, treatments were performed in the presence of 10% FBS-DMEM: F12 medium for 24 hours. Then, cells were collected and counted by using a flow cytometer. An additional experimental control (control 1) was utilized (and compared to control 2) to exclude any potential interference triggered by FBS replacement on the cell number. Our results showed a significant reduction (***P < 0.001) in the total cell count with increasing concentrations of the guarana as well as combinations of guarana with caffeine and/or taurine (Figure 11(a)). A similar pattern was observed in caffeine-treated cells, especially with 2 mg/mL (***P < 0.001). Taurine significantly reduced the total number of cells at concentrations of 1 and 4 mg/mL (***P < 0.001 and **P < 0.01, resp.) but not 16 mg/mL. Representative PCM micrographs of the cells were taken before proceeding with flow cytometric analysis (Figure 11(b)). Signs of generalized cell death were observed in guarana-treated cells (Figures 11(b)(3)–11(b)(5)) or its combinations with caffeine and/or taurine (Figures 11(b)(12), 11(b)(13), and 11(b)(16)). In these cultures, cells seem to have lost the typical neuronal phenotype and characteristic neuritic processes of SH-SY5Y cells, became rounded, and, thereafter, lost their viability. Milder signs of the same morphological events were also observed in cells treated with 2 mg/mL of caffeine (Figure 11(b)(8)). On the contrary, taurine (1, 4, and 16 mg/mL) did not induce any morphological sign of cell death after 24 hours of treatment (Figures 11(b)(9)–11(b)(11)).

**Figure 12:**
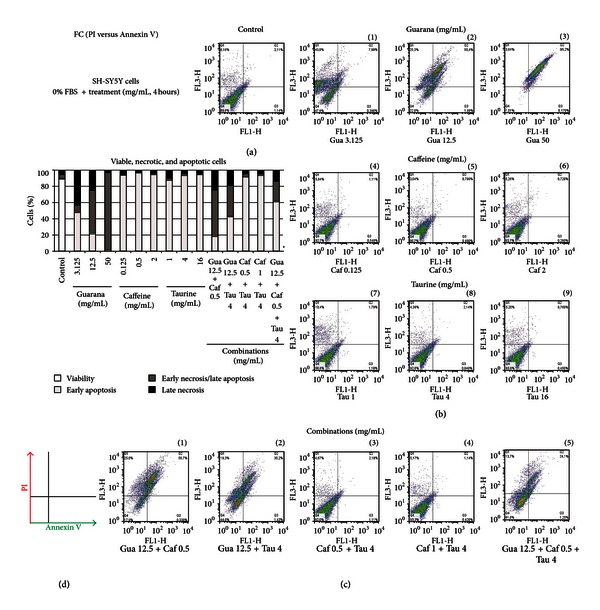
Analysis of drug-induced effects on cellular viability. Flow cytometric analysis (FC) of the fraction (%) of viable, apoptotic, and necrotic human neuronal SH-SY5Y cells by propidium iodide (PI) versus Annexin V, after treatments with different concentrations of guarana (3.125, 12.5, and 50 mg/mL), caffeine (0.125, 0.5, and 2 mg/mL), taurine (1, 4, and 16 mg/mL), and their combinations for 4 hours.

In order to assess the type of cell death induced by these major components of EDs, flow cytometric analyses with Annexin V and PI were performed after 4 hours of treatment. Both qualitative and quantitative data were obtained, and the fraction (in %) of live (Q4), early apoptotic (Q3), late apoptotic (Q2), and necrotic (Q1) cells was given after counting 10000 cells per case (Figure 12). Our results show that treatments with increasing concentrations of guarana and its combinations with caffeine and/or taurine decrease the percentage of viable cells (Figures 12(b)(1)–12(b)(3), 12(c)(1), 12(c)(2), and 12(c)(5)) when compared to control cells (untreated) (Figure 12(a)). Interestingly, it seems that higher concentrations of guarana (12.5 and 50 mg/mL) enrich the fraction of cells undergoing late apoptosis (from 7.98 to 95.2%, resp.) from the total percentage of unviable cells (Figure 12(d)).

### 3.6. *In Silico* Development and Topological Analysis of a Network Model for the Interactions of ED Components through Redox/Nitric Oxide (NO) and Apoptotic Pathways (MEDRI Model) Highlight “Hydroxyl Radicals” as the Node with the Highest Stress

Many of the components present in EDs (e.g., taurine, guarana, or carnitine) are known to exert antioxidant activity in different biological systems [25–27]. Based on this fact and the previous evidence of apoptosis and/or neurite degeneration, we decided to establish an *in silico* evaluation of the general landscape of interactions between ED components as well as redox/NO and apoptosis-related proteins, by using systems biology tools. For instance, based on the network centralities (e.g., connectivity and stress), it is virtually possible to identify “essential” nodes in a newly developed interaction network [29, 30]. Targeting essential nodes with high centrality values can considerably disrupt the whole network integrity. Moreover, high stress value for a given node can be an indicator of relevance of a protein/compound within a biological network, and it is commonly used tool in computational biology-based studies [31, 32].

**Figure 13:**
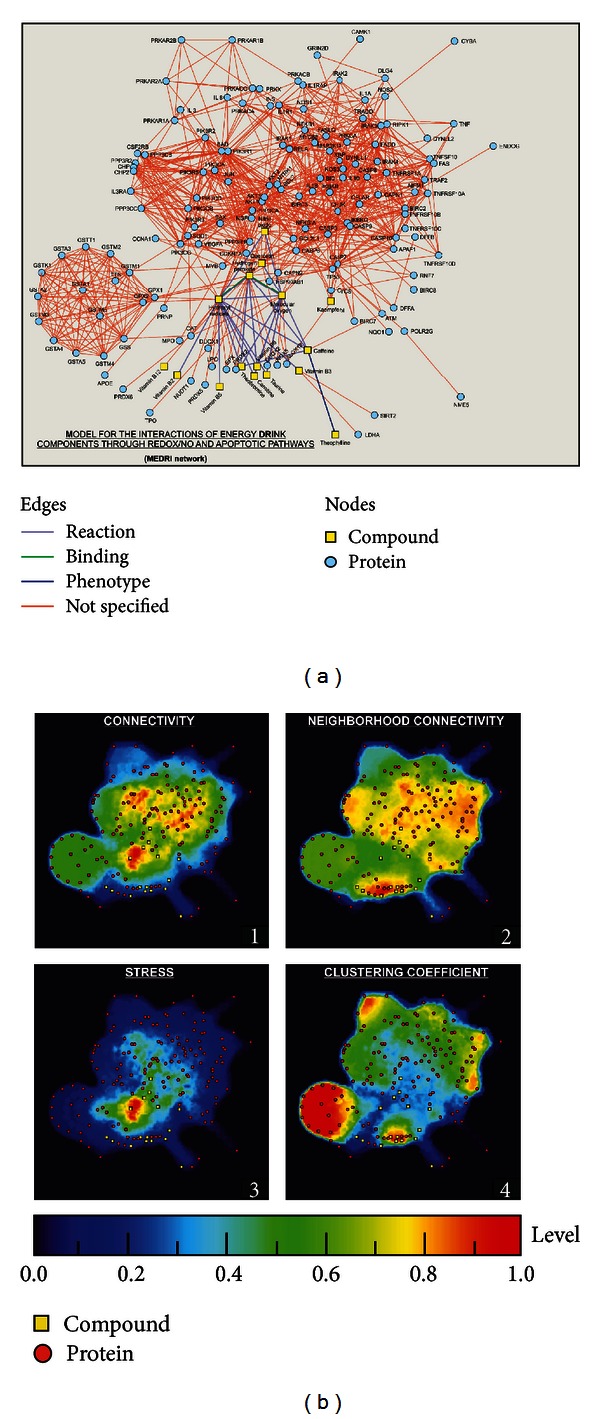
Model for the interactions of ED components through redox/NO and apoptotic pathways. (a) *In silico* analysis of the interactions of ED components through redox and NO pathways, based on experimental data and database, gave rise to a network model (MEDRI) where exactly 16 compounds (12 ED components, hydrogen peroxide, hydroxyl radicals, molecular oxygen, and nitric oxide) and 144 proteins (87 apoptosis-related and 57 redox/NO-related proteins) interconnected, considering their potential interaction through either “activation,” “inhibition,” “catalysis,” “binding,” or “reaction.” (b) Analysis of the topological network properties by Cytoscape and its 2D projection by ViaComplex Software show in a color-grading representation the areas of maximum values (the closer to red color the higher value) of connectivity, neighborhood connectivity, stress, and clustering coefficient of each node.

 Our *in silico* analysis gave rise to the MEDRI network model (Figure 13(a)) where 87 apoptosis-related and 57 redox/NO-related proteins interconnected with 12 ED components linked through 4 additional compounds: hydroxyl radicals, molecular oxygen, hydrogen peroxide, and nitric oxide. 

 Topological network properties (connectivity, neighborhood connectivity, stress, and clustering coefficient) of the nodes (proteins and compounds) highlighted “hydroxyl radicals” as a highly interconnected node with the highest value of stress centrality in the interaction model (MEDRI) (Figure 13(b) and Supporting Information Table  S4). 

## 4. Discussion

Even though caffeine is considered the main active ingredient in EDs, the presence of other substances such as taurine, B vitamins, carnitine, and diverse herbal derivatives should be considered, since both acute and long-term effects of its combined consumption with caffeine and related methylxanthines are not well known. 

 Here, we used human neuronal SH-SY5Y cells to perform morphological and cytotoxic evaluations of the most common components of EDs (caffeine, taurine, and guarana), alone or in combination. SH-SY5Y cell line is an immortalized cell culture system with obvious limitations for data interpretation, mainly due to the differences in cell cycle state and expression levels of neuronal markers that are observed in mature neurons. However, this and other catecholaminergic cell lines are widely used for toxicological studies at the cellular and molecular levels, due to inherent advantages in relation to primary neuronal culture systems, for instance, the human origin of these cells, high cellular homogeneity (rodent primary dopaminergic culture systems present only 5% of tyrosine hydroxylase-positive cells), and the presence of many biochemical and functional characteristics of neurons that are easily measurable and detectable, such as neurite extension, *β*-III-tubulin, and tyrosine hydroxylase expression, as well as catecholamine production and secretion. Moreover, these cells exhibit cellular responses to cytotoxic compounds that are similar to those found in human primary cultures [10, 33–35].

In general, ROS are able to react with diverse cellular components (e.g., DNA, carbohydrates, proteins, and lipids) in a destructive way. These entities include free radicals (superoxide, nitric oxide, and hydroxyl radicals) as well as other molecular species (hydrogen peroxide and peroxynitrite). In neurodegenerative processes, it is believed that neurological impairment is intrinsically linked to ROS-triggered neuronal apoptosis [28]. Here, *in vitro* morphological analyses showed clear signs of apoptosis, such membrane blebbing, cell shrinkage, and cleaved caspase-3 positive cells in guarana-treated cells (12.5–50 mg/mL) after 2–4 hours of treatment (Figure 2). Then, we characterized the complete redox profile of SH-SY5Y cells after treatments with ED components, in order to see how changes in ROS, nonenzymatic and enzymatic antioxidant activity, and oxidative alterations in proteins and/or lipids may correlate with potential changes in the cellular viability. Our analysis of different oxidative stress-related parameters showed strong antioxidant effects induced in SH-SY5Y cells when treated with ED components, especially with guarana and its combinations with caffeine and/or taurine, which are consistent with the strong *in vitro* antioxidant potential exerted by guarana and observed in TRAP and TAR measurements (Figure 7). The decrease of the basal levels of free radical generation after these treatments (Figure 8(b)) may result in down-regulation of the cellular enzymatic antioxidant defense (e.g., SOD and CAT activities) (Figure 10). The concomitant loss of viability and induction of apoptotic parameters in cells under the same treatments (Figures 11 and 12) indicate that this strong antioxidant effect is responsible, at least in part, for the *in vitro* cytotoxic effect exerted by ED components.

Although induction of cellular necrosis and apoptosis is generally associated with prooxidant conditions leading to oxidative stress, it must be pointed out that physiological ROS levels exert essential roles in the maintenance of cellular homeostasis. The regulation of protein activity by oxidation and reduction of lateral chains in certain residues has been recognized as one of the most important mechanisms of cell function regulation, together with protein phosphorylation [28]. Since ROS regulate the mitogen-activated protein kinase (MAPK) and the phosphoinositide 3-kinase (PI3 K) [36], it is very likely that excessive intracellular ROS scavenging after exposure to drug/s with high antioxidant potential (alone or in combinations) may disrupt the cellular redox homeostasis and, thus, proliferation and survival-related signaling pathways. Such a concept, where a strong antioxidant-derived redox imbalance can be as dangerous as oxidative stress, is already known in the literature as “antioxidative stress” [37] (Figure 13). As a matter of fact, *in vitro* induction of cellular apoptosis associated with drug antioxidant activity is already known for a number of substances (e.g., flavonoids, polyphenols, and saponins) [38–40]. Therefore, the concept of antioxidative stress would be particularly relevant in the case of excessive consumption of nutritional supplements or beverages (like EDs), where diverse substances with antioxidant potential are mixed together at relatively high concentrations. For instance, the Brazilian native plant, guarana, traditionally used as stimulant and aphrodisiac [41], has already been shown to exert antibacterial and antioxidant activities in 3T3-L1 cells [25]. One of its major components, caffeine, is an antagonist of adenosine receptors able to inhibit phosphodiesterase (PDE), GABA receptor-mediated effects [42, 43], and has been suggested to have a role in preventing age-associated decline in cognitive function after its chronic ingestion, through the protection of the antioxidant system *in vivo* [44]. Finally, taurine (2-aminoethanesulfonic acid) is a sulfur amino acid commonly found in electrically excitable tissues, such as heart or brain [45], which is known to exert other diverse effects in organs and tissues like modulation of calcium levels, stabilization of membranes, and antioxidant and antiapoptotic effects able to improve wound healing [27, 45–47]. 

In this study, we postulated that combined effect of excessive depletion of ROS-mediated cellular signaling together with caffeine-mediated induction of apoptosis [48] would lead SH-SY5Y cells to lose their viability and undergo apoptosis after guarana treatments, alone or in combination with additional caffeine and/or taurine doses. In fact, induction of apoptosis in SH-SY5Y cells by other classical antioxidant compounds (e.g., sodium ascorbate or vitamin C) is already known [49].

It has been described that 24-hour treatment with low concentrations of caffeine induces p53-dependent apoptosis in JB6 cells through the Bax and caspase-3 pathways [48]. Additionally, it is known that 1–10 mM of caffeine triggers calcium release from intracellular stores through a caffeine-sensitive ryanodine receptor, and such increase could lead to cell death in the central nervous system [50, 51]. Furthermore, calcium itself could directly activate caspases (e.g., caspase-3) or even proteases, having a role in the degeneration of neurofilaments [52–54]. In our study, 24-hour treatment with 2 mg/mL of caffeine significantly decreased the total cell number (Figure 11(a)) and induced typical morphological signs of decreased viability *in vitro* (Figure 11(b)(8)). Our data also showed signs of neurite degeneration in the form of swellings at various segments in a beaded or pearl chain-like structure together with neurite fragmentation in guarana-treated cells (12.5–50 mg/mL; providing approximately 0.5 and 2 mg/mL of caffeine, resp.), as well as cells treated with combinations of guarana with caffeine and/or taurine (Figures 2, 5, and 8(a)). Caffeine-treated cells did not exhibit fragmented neurites, but the presence of neuritic pearl chain-like structures was indeed abundant, especially with 2 mg/mL (Figures 3 and 5). These structures are similar to those found in a wide variety of neurological diseases as axonal defects (and impaired axonal transport) in the form of swellings that accumulate excessive amounts of proteins, organelles, and vesicles [55]. In contrast, healthy neuritic processes were observed in taurine-treated cells (1 to 16 mg/mL) as shown by PCM, SEM, and *β*-III-tubulin immunofluorescence staining (Figures 4 and 6) and confirmed after long-term taurine treatments (24 hours) (Figure 11(b)). Interestingly, *in vitro* taurine-derived neuronal differentiation and its remarkable neuroprotective role in cerebellar granule cells have already been demonstrated and the mechanism would involve the regulation of cytoplasmic free calcium and intramitochondrial calcium homeostasis [56, 57]. This might explain the apparent decrease in percentage of late apoptotic cells in taurine combination with guarana when compared to guarana-treated cells and the absence of neuritic swellings in cells treated with 0.5 mg/mL of caffeine together with 4 mg/mL of taurine (Figure 8(a)). 

Perhaps, the large number and mixture of active compounds in EDs, at relatively high concentrations, may represent the biggest obstacle for designing efficient experimental plans to determine any potential toxicity at the molecular level of these beverages. Nowadays, systems biology/pharmacology/toxicology offers a number of computational tools that allow researchers to work with large amounts of experimental knowledge and databases for elaborating simulations or supporting hypotheses, that can be later confirmed in other models (e.g., *in vivo* or *in vitro*) [11, 31, 58, 59]. 

In this study, our strategy consisted in developing a network model of interactions between ED components (Supporting Information Table  S3) and proteins belonging to the routes of interest, in this case, redox/NO and apoptotic pathways. 

Text mining or text analytics for constructing our *in silico* model were avoided, which means that, instead of discussions or hypotheses from the literature, exclusively experimental data and databases were used as sources of information (from STRING 9.0 and STITCH 3.0) for constructing the MEDRI network. Interestingly enough, proteins of redox/NO and apoptotic pathways interconnected with ED components (Figure 13(a)) through reaction with highly stressed and connected (topologically speaking) “hydroxyl radicals” and “hydrogen peroxide” nodes (Supporting Information Table  S4 and Figures 13(b)(1) and 13(b)(3)) in this model (Figure 13(a)), strongly supporting a redox component in the modulation of cell survival/death by ED components.

## 5. Conclusion

**Figure 14:**
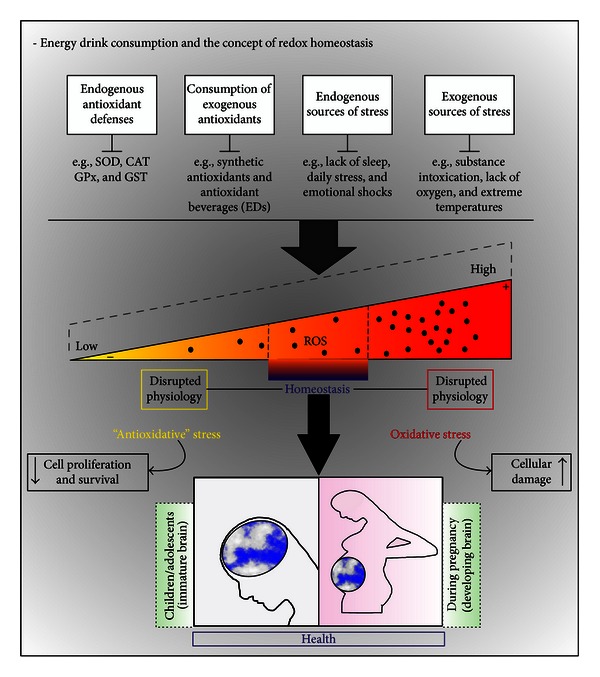
EDs and the concept of redox homeostasis. In an individual organism, homeostasis is required for healthy development and normal tissue functioning. In our daily life, one can be exposed to diverse sources of stress (endogenous and/or exogenous) able to impair the physiology of developing brains (e.g., during pregnancy, children, or adolescents). In these cases, our antioxidant defenses should maintain the homeostatic levels of ROS in order to avoid an oxidative stress scenario. Paradoxically, the relevant role of ROS in regulating cell proliferation and survival signaling pathways is already known. Therefore, an excessive antioxidant-derived redox imbalance (“antioxidative” stress) would also impair homeostasis, becoming as dangerous as oxidative stress itself. In our study, we show *in vitro* evidence that highlights the danger of energy drink consumption and how their potential cytotoxicity could be partially attributed to the excessive depletion of intracellular ROS and disruption of redox homeostasis, due to the combination of multiple components with antioxidant activity in these beverages.

Despite the limitations of SH-SY5Y cells as an *in vitro* model, our results suggest that guarana and caffeine, alone or in combination with taurine, may exert neurotoxicological effects in part by disruption of redox homeostasis (Figure 14), which is also consistent with the data obtained in the *in silico* approach (Figure 13).

We believe that our results highlight the need for further *in vivo* and epidemiological studies to unravel the potential for serious adverse health effects of ED consumption.

## Supplementary Material

Supplementary materials contain the identifiers of proteins (Ensembl) and compounds (CID) contributing to the *in silico* network model of interactions of energy drink components through REDOX/NO and apoptotic pathways (MEDRI network), together with the network topology values for clustering coefficient, connectivity, neighborhood connectivity, and stress.

## Abbreviations

EDs: Energy drinks
Gua: Guarana
Caf: Caffeine
Tau: Taurine
PCM: Phase contrast microscopy
SEM: Scanning electron microscopy
CM: Confocal microscopy
FC: Flow cytometry.

## References

[1] Reissig CJ, Strain EC, Griffiths RR (2009). Caffeinated energy drinks: a growing problem. *Drug and Alcohol Dependence*.

[2] Seifert SM, Schaechter JL, Hershorin ER, Lipshultz SE (2011). Health effects of energy drinks on children, adolescents, and young adults. *Pediatrics*.

[3] Gunja N, Brown JA (2012). Energy drinks: health risks and toxicity. *Medical Journal of Australia*.

[4] Higgins JP, Tuttle TD, Higgins CL (2010). Energy beverages: content and safety. *Mayo Clinic Proceedings*.

[5] Alford C, Cox H, Wescott R (2001). The effects of Red Bull Energy Drink on human performance and mood. *Amino Acids*.

[6] Marczinski CA, Fillmore MT, Bardgett ME, Howard MA (2011). Effects of energy drinks mixed with alcohol on behavioral control: risks for college students consuming trendy cocktails. *Alcoholism: Clinical and Experimental Research*.

[7] Iyadurai SJP, Chung SS (2007). New-onset seizures in adults: possible association with consumption of popular energy drinks. *Epilepsy and Behavior*.

[8] Arria AM, O’Brien M (2011). The “high” risk of energy drinks. *The Journal of the American Medical Association*.

[9] Temple JL (2009). Caffeine use in children: what we know, what we have left to learn, and why we should worry. *Neuroscience and Biobehavioral Reviews*.

[10] Agholme L, Lindström T, Kgedal K, Marcusson J, Hallbeck M (2010). An in vitro model for neuroscience: differentiation of SH-SY5Y cells into cells with morphological and biochemical characteristics of mature neurons. *Journal of Alzheimer’s Disease*.

[11] Rabelo TK, Zeidán-Chuliá F, Vasques LM (2012). Redox characterization of usnic acid and its cytotoxic effect on human neuron-like cells (SH-SY5Y). *Toxicology in Vitro*.

[12] da Frota Junior ML, Pires AS, Zeidán-Chuliá F (2011). In vitro optimization of retinoic acid-induced neuritogenesis and TH endogenous expression in human SH-SY5Y neuroblastoma cells by the antioxidant Trolox. *Molecular and Cellular Biochemistry*.

[13] Habig WH, Pabst MJ, Jakoby WB (1974). Glutathione S-transferases. The first enzymatic step in mercapturic acid formation. *Journal of Biological Chemistry*.

[14] Ellman GL (1959). Tissue sulfhydryl groups. *Archives of Biochemistry and Biophysics*.

[15] Szklarczyk D, Franceschini A, Kuhn M (2011). The STRING database in 2011: functional interaction networks of proteins, globally integrated and scored. *Nucleic Acids Res*.

[16] Wain HM, Lush MJ, Ducluzeau F, Khodiyar VK, Povey S (2004). Genew: the human gene nomenclature database, 2004 updates. *Nucleic Acids Research*.

[17] Birney E, Andrews D, Caccamo M (2006). Ensembl 2006. *Nucleic Acids Research*.

[18] Kuhn M, von Mering C, Campillos M, Jensen LJ, Bork P (2008). STITCH: interaction networks of chemicals and proteins. *Nucleic Acids Research*.

[19] Hooper SD, Bork P (2005). Medusa: a simple tool for interaction graph analysis. *Bioinformatics*.

[20] Smoot ME, Ono K, Ruscheinski J, Wang PL, Ideker T (2011). Cytoscape 2.8: new features for data integration and network visualization. *Bioinformatics*.

[21] Castro MAA, Rybarczyk Filho JL, Dalmolin RJS (2009). ViaComplex: software for landscape analysis of gene expression networks in genomic context. *Bioinformatics*.

[22] Lowry OH, Rosebrough NJ, Farr AL, Randal RJ (1951). Protein measurement with the Folin phenol reagent. *The Journal of Biological Chemistry*.

[23] Santa Maria A, Lopez A, Diaz MM, Muñoz-Mingarro D, Pozuelo JM (1998). Evaluation of the toxicity of guarana with in vitro bioassays. *Ecotoxicology and Environmental Safety*.

[24] Bydlowski SP, Yunker RL, Subbiah MTR (1988). A novel property of an aqueous guarana extract (Paullinia cupana): inhibition of platelet aggregation in vitro and in vivo. *Brazilian Journal of Medical and Biological Research*.

[25] Basile A, Ferrara L, Del Pezzo M (2005). Antibacterial and antioxidant activities of ethanol extract from Paullinia cupana Mart. *Journal of Ethnopharmacology*.

[26] Gülçin I (2006). Antioxidant and antiradical activities of L-carnitine. *Life Sciences*.

[27] Gültekin SE, Sengüven B, Sofuoğlu A, Taner L, Koch M (2012). Effect of the topical use of the antioxidant taurine on the two basement membrane proteins of regenerating oral gingival epithelium. *Journal of Periodontology*.

[28] Halliwel B, Gutteridge JMC (2005). *Free Radicals in Biology and Medicine*.

[29] Wuchty S, Stadler PF (2003). Centers of complex networks. *Journal of Theoretical Biology*.

[30] Estrada E (2006). Virtual identification of essential proteins within the protein interaction network of yeast. *Proteomics*.

[31] Zeidán-Chuliá F, Rybarczyk-Filho JL, Gursoy M (2012). Bioinformatical and in vitro approaches to essential oil-induced matrix metalloproteinase inhibition. *Pharmaceutical Biology*.

[32] Rosado JO, Henriques JP, Bonatto D (2011). A systems pharmacology analysis of major chemotherapy combination regimens used in gastric cancer treatment: predicting potential new protein targets and drugs. *Current Cancer Drug Targets*.

[33] Biedler JL, Helson L, Spengler BA (1973). Morphology and growth, tumorigenicity, and cytogenetics of human neuroblastoma cells in continuous culture. *Cancer Research*.

[34] Sanfeliu C, Cristòfol R, Torán N, Rodríguez-Farré E, Kim SU (1999). Use of human central nervous system cell cultures in neurotoxicity testing. *Toxicology in Vitro*.

[35] Morales-Hernández A, Sánchez-Martín FJ, Hortigón-Vinagre MP, Henao F, Merino JM (2012). 2, 3, 7, 8-tetrachlorodibenzo-p-dioxin induces apoptosis by disruption of intracellular calcium homeostasis in human neuronal cell line SHSY5Y. *Apoptosis*.

[36] Ray PD, Huang BW, Tsuji Y (2012). Reactive oxygen species (ROS) homeostasis and redox regulation in cellular signaling. *Cellular Signalling*.

[37] Poljsak B, Milisav I (2012). The neglected significance of ‘antioxidative stress’. *Oxidative Medicine and Cellular Longevity*.

[38] Seeram NP, Adams LS, Henning SM (2005). In vitro antiproliferative, apoptotic and antioxidant activities of punicalagin, ellagic acid and a total pomegranate tannin extract are enhanced in combination with other polyphenols as found in pomegranate juice. *Journal of Nutritional Biochemistry*.

[39] Hsu CL, Yen GC (2006). Induction of cell apoptosis in 3T3-L1 pre-adipocytes by flavonoids is associated with their antioxidant activity. *Molecular Nutrition and Food Research*.

[40] He NW, Zhao Y, Guo L, Shang J, Yang XB (2012). Antioxidant, antiproliferative, and pro-apoptotic activities of a saponin extract derived from the roots of Panax notoginseng (Burk.) F.H. Chen. *Journal of Medicinal Food*.

[41] Fukumasu H, Avanzo JL, Nagamine MK, Barbuto JA, Rao KV, Dagli MLZ (2008). Paullinia cupana Mart var. sorbilis, guaraná, reduces cell proliferation and increases apoptosis of B16/F10 melanoma lung metastases in mice. *Brazilian Journal of Medical and Biological Research*.

[42] Howell LL (1993). Comparative effects of caffeine and selective phosphodiesterase inhibitors on respiration and behavior in rhesus monkeys. *Journal of Pharmacology and Experimental Therapeutics*.

[43] Lopez F, Miller LG, Greenblatt DJ, Kaplan GB, Shader RI (1989). Interaction of caffeine with the GABA(A) receptor complex: alterations in receptor function but not ligand binding. *European Journal of Pharmacology*.

[44] Abreu RV, Silva-Oliveira EM, Moraes MF, Pereira GS, Moraes-Santos T (2011). Chronic coffee and caffeine ingestion effects on the cognitive function and antioxidant system of rat brains. *Pharmacology Biochemistry and Behavior*.

[45] Huxtable RJ (1992). Physiological actions of taurine. *Physiological Reviews*.

[46] Değim Z, Celebi N, Sayan H, Babül A, Erdoğan D, Take G (2002). An investigation on skin wound healing in mice with a taurine-chitosan gel formulation. *Amino Acids*.

[47] Giriş M, Depboylu B, Doğru-Abbasoğlu S (2008). Effect of taurine on oxidative stress and apoptosis-related protein expression in trinitrobenzene sulphonic acid-induced colitis. *Clinical and Experimental Immunology*.

[48] He Z, Ma WY, Hashimoto T, Bode AM, Yang CS, Dong Z (2003). Induction of apoptosis by caffeine is mediated by the p53, Bax, and caspase 3 pathways. *Cancer Research*.

[49] Carosio R, Zuccari G, Orienti I, Mangraviti S, Montaldo PG (2007). Sodium ascorbate induces apoptosis in neuroblastoma cell lines by interfering with iron uptake. *Molecular Cancer*.

[50] Orrenius S, Nicotera P (1994). The calcium ion and cell death. *Journal of Neural Transmission, Supplement*.

[51] Jang MH, Shin MC, Kang IS (2002). Caffeine induces apoptosis in human neuroblastoma cell line SK-N-MC. *Journal of Korean Medical Science*.

[52] Nordin-Andersson M, Forsby A, Heldring N, DeJongh J, Kjellstrand P, Walum E (1998). Neurite degeneration in differentiated human neuroblastoma cells. *Toxicology in Vitro*.

[53] Pörn-Ares MI, Ares MPS, Orrenius S (1998). Calcium signalling and the regulation of apoptosis. *Toxicology in Vitro*.

[54] Pelletier M, Oliver L, Meflah K, Vallette FM (2005). Caspase-3 can be pseudo-activated by a Ca^2+^-dependent proteolysis at a non-canonical site. *FEBS Letters*.

[55] Stokin GB, Lillo C, Falzone TL (2005). Axonopathy and transport deficits early in the pathogenesis of Alzheimer’s diseases. *Science*.

[56] Spoerri PE, Caple CG, Roisen FJ (1990). Taurine-induced neuronal differentiation: the influence of calcium and the ganglioside GM1. *International Journal of Developmental Neuroscience*.

[57] El Idrissi A (2008). Taurine increases mitochondrial buffering of calcium: role in neuroprotection. *Amino Acids*.

[58] Zeidán-Chuliá F, Rybarczyk-Filho JL, Salmina AB, de Oliveira BH, Noda M, Moreira JC (2013). Exploring the multifactorial nature of autism through computational systems biology: calcium and the Rho GTPase RAC1 under the spotlight. *NeuroMolecular Medicine*.

[59] Zeidán-Chuliá F, Neves de Oliveira BH, Gursoy M (2013). MMP-REDOX/NO interplay in periodontitis and its inhibition with Satureja hortensis L. essential oil. *Chemistry & Biodiversity*.

