# Supplementary material for: Major Components of Energy Drinks (Caffeine, Taurine, and Guarana) Exert Cytotoxic Effects on Human Neuronal SH-SY5Y Cells by Decreasing Reactive Oxygen Species Production
Source: Oxid Med Cell Longev. 2013 May 22;2013:791795. doi: 10.1155/2013/791795 (PMC3674721; doi:10.1155/2013/791795)
Supplement: Supplementary file 1 — Supplementary materials contain the identifiers of proteins (Ensembl) and compounds (CID) contributing to the in silico network model of interactions of energy drink components through REDOX/NO and apoptotic pathways (MEDRI network), together with the network topology values for clustering coefficient, connectivity, neighborhood connectivity, and stress. [file 791795.f1.zip › Table S1.docx]

**Supporting Information Table S1.** Ensembl protein identifiers of the redox/nitric oxide (NO)-related proteins belonging to the MEDRI model.

| **REDOX/NO** | | | | | |
| --- | --- | --- | --- | --- | --- |
| Gene symbol | ENSEMBL ID | Gene symbol | ENSEMBL ID | Gene symbol | ENSEMBL ID |
| ALOX12 | ENSP00000251535 | GSTA3 | ENSP00000211122 | NME5 | ENSP00000265191 |
| APOE | ENSP00000252486 | GSTA4 | ENSP00000359998 | NOS1 | ENSP00000320758 |
| CAMK1 | ENSP00000256460 | GSTA5 | ENSP00000284562 | NOS2 | ENSP00000327251 |
| CAT | ENSP00000241052 | GSTK1 | ENSP00000367415 | NOS3 | ENSP00000297494 |
| CCNA1 | ENSP00000255465 | GSTM1 | ENSP00000311469 | NOX5 | ENSP00000260364 |
| CDKN1A | ENSP00000244741 | GSTM2 | ENSP00000241337 | NQO1 | ENSP00000319788 |
| CYBA | ENSP00000261623 | GSTM3 | ENSP00000256594 | NUDT1 | ENSP00000339503 |
| DLG4 | ENSP00000293813 | GSTM4 | ENSP00000358851 | POLR2G | ENSP00000301788 |
| DUOX1 | ENSP00000317997 | GSTM5 | ENSP00000256593 | PRDX2 | ENSP00000301522 |
| DUOX2 | ENSP00000267837 | GSTT1 | ENSP00000401632 | PRDX5 | ENSP00000265462 |
| DYNLL1 | ENSP00000242577 | HSP90AB1 | ENSP00000325875 | PRDX6 | ENSP00000342026 |
| DYNLL2 | ENSP00000240343 | IL10 | ENSP00000412237 | PRKCA | ENSP00000284384 |
| EPX | ENSP00000225371 | IL8 | ENSP00000306512 | PRNP | ENSP00000368748 |
| GPX1 | ENSP00000407375 | INS | ENSP00000348986 | RNF7 | ENSP00000273480 |
| GPX2 | ENSP00000374265 | JUN | ENSP00000360266 | SIRT2 | ENSP00000249396 |
| GRIN2D | ENSP00000263269 | LDHA | ENSP00000395337 | SOD1 | ENSP00000270142 |
| GSS | ENSP00000216951 | LPO | ENSP00000262290 | TPO | ENSP00000329869 |
| GSTA1 | ENSP00000335620 | MPO | ENSP00000225275 | TTN | ENSP00000348444 |
| GSTA2 | ENSP00000420168 | MYB | ENSP00000339992 | VEGFA | ENSP00000361125 |
