# Supplementary material for: Major Components of Energy Drinks (Caffeine, Taurine, and Guarana) Exert Cytotoxic Effects on Human Neuronal SH-SY5Y Cells by Decreasing Reactive Oxygen Species Production
Source: Oxid Med Cell Longev. 2013 May 22;2013:791795. doi: 10.1155/2013/791795 (PMC3674721; doi:10.1155/2013/791795)
Supplement: Supplementary file 1 — Supplementary materials contain the identifiers of proteins (Ensembl) and compounds (CID) contributing to the in silico network model of interactions of energy drink components through REDOX/NO and apoptotic pathways (MEDRI network), together with the network topology values for clustering coefficient, connectivity, neighborhood connectivity, and stress. [file 791795.f1.zip › Table S2.docx]

**Supporting Information Table S2.** Ensembl protein identifiers of the apoptosis-related proteins belonging to the MEDRI model.

| **APOPTOSIS** | | | | | |
| --- | --- | --- | --- | --- | --- |
| Gene symbol | ENSEMBL ID | Gene symbol | ENSEMBL ID | Gene symbol | ENSEMBL ID |
| AIFM1 | ENSP00000287295 | DFFA | ENSP00000366237 | PIK3R2 | ENSP00000222254 |
| AKT1 | ENSP00000270202 | DFFB | ENSP00000367454 | PIK3R3 | ENSP00000262741 |
| AKT2 | ENSP00000375892 | ENDOG | ENSP00000361725 | PIK3R5 | ENSP00000269300 |
| AKT3 | ENSP00000263826 | FADD | ENSP00000301838 | PPP3CA | ENSP00000378323 |
| APAF1 | ENSP00000353059 | FAS | ENSP00000347979 | PPP3CB | ENSP00000378306 |
| ATM | ENSP00000278616 | FASLG | ENSP00000356694 | PPP3CC | ENSP00000240139 |
| BAD | ENSP00000309103 | IKBKB | ENSP00000339151 | PPP3R1 | ENSP00000234310 |
| BAX | ENSP00000293288 | IKBKG | ENSP00000358622 | PPP3R2 | ENSP00000363939 |
| BCL2 | ENSP00000329623 | IL1A | ENSP00000263339 | PRKACA | ENSP00000309591 |
| BCL2L1 | ENSP00000302564 | IL1B | ENSP00000263341 | PRKACB | ENSP00000359719 |
| BID | ENSP00000318822 | IL1R1 | ENSP00000233946 | PRKACG | ENSP00000366488 |
| BIRC2 | ENSP00000227758 | IL1RAP | ENSP00000072516 | PRKAR1A | ENSP00000351410 |
| BIRC3 | ENSP00000263464 | IL3 | ENSP00000296870 | PRKAR1B | ENSP00000353415 |
| BIRC7 | ENSP00000217169 | IL3RA | ENSP00000327890 | PRKAR2A | ENSP00000265563 |
| BIRC8 | ENSP00000412957 | IRAK1 | ENSP00000358997 | PRKAR2B | ENSP00000265717 |
| CAPN1 | ENSP00000279247 | IRAK2 | ENSP00000256458 | PRKX | ENSP00000262848 |
| CAPN2 | ENSP00000295006 | IRAK3 | ENSP00000261233 | RELA | ENSP00000384273 |
| CASP10 | ENSP00000286186 | IRAK4 | ENSP00000349096 | RIPK1 | ENSP00000259808 |
| CASP3 | ENSP00000311032 | MAP3K14 | ENSP00000342059 | TNF | ENSP00000392858 |
| CASP6 | ENSP00000265164 | MYD88 | ENSP00000379625 | TNFRSF10A | ENSP00000221132 |
| CASP7 | ENSP00000298700 | NFKB1 | ENSP00000226574 | TNFRSF10B | ENSP00000276431 |
| CASP8 | ENSP00000351273 | NFKBIA | ENSP00000216797 | TNFRSF10C | ENSP00000310908 |
| CASP9 | ENSP00000330237 | NGF | ENSP00000358525 | TNFRSF10D | ENSP00000310263 |
| CFLAR | ENSP00000312455 | NTRK1 | ENSP00000351486 | TNFRSF1A | ENSP00000162749 |
| CHP | ENSP00000335632 | PIK3CA | ENSP00000263967 | TNFSF10 | ENSP00000241261 |
| CHP2 | ENSP00000300113 | PIK3CB | ENSP00000289153 | TP53 | ENSP00000269305 |
| CHUK | ENSP00000359424 | PIK3CD | ENSP00000366563 | TRADD | ENSP00000341268 |
| CSF2RB | ENSP00000384053 | PIK3CG | ENSP00000352121 | TRAF2 | ENSP00000247668 |
| CYCS | ENSP00000307786 | PIK3R1 | ENSP00000274335 | XIAP | ENSP00000347858 |
