# Supplementary material for: Major Components of Energy Drinks (Caffeine, Taurine, and Guarana) Exert Cytotoxic Effects on Human Neuronal SH-SY5Y Cells by Decreasing Reactive Oxygen Species Production
Source: Oxid Med Cell Longev. 2013 May 22;2013:791795. doi: 10.1155/2013/791795 (PMC3674721; doi:10.1155/2013/791795)
Supplement: Supplementary file 1 — Supplementary materials contain the identifiers of proteins (Ensembl) and compounds (CID) contributing to the in silico network model of interactions of energy drink components through REDOX/NO and apoptotic pathways (MEDRI network), together with the network topology values for clustering coefficient, connectivity, neighborhood connectivity, and stress. [file 791795.f1.zip › Table S3.docx]

**Supporting Information Table S3.** Compounds and compound identifiers belonging to the MEDRI model.

| **COMPOUNDS** | |
| --- | --- |
| Compound name | Compound ID |
| Caffeine | CID000002519 |
| Carnitine | CID000000085 |
| Hydrogen peroxide | CID000000784 |
| Hydroxyl radicals | CID000000961 |
| Kaempferol | CID005280863 |
| Molecular oxygen | CID000000977 |
| Nitric oxide | CID000145068 |
| Quercetin | CID005280343 |
| Taurine | CID000001123 |
| Theobromine | CID000005429 |
| Theophylline | CID000002153 |
| Vitamin B2 | CID000493570 |
| Vitamin B3 | CID000000936 |
| Vitamin B5 | CID000006613 |
| Vitamin B6 | CID000001054 |
| Vitamin B12 | CID016686079 |
