# Supplementary material for: Major Components of Energy Drinks (Caffeine, Taurine, and Guarana) Exert Cytotoxic Effects on Human Neuronal SH-SY5Y Cells by Decreasing Reactive Oxygen Species Production
Source: Oxid Med Cell Longev. 2013 May 22;2013:791795. doi: 10.1155/2013/791795 (PMC3674721; doi:10.1155/2013/791795)
Supplement: Supplementary file 1 — Supplementary materials contain the identifiers of proteins (Ensembl) and compounds (CID) contributing to the in silico network model of interactions of energy drink components through REDOX/NO and apoptotic pathways (MEDRI network), together with the network topology values for clustering coefficient, connectivity, neighborhood connectivity, and stress. [file 791795.f1.zip › Table S4.docx]

#### Supporting Information Table S4. Values for clustering coefficient, connectivity, neighborhood connectivity, and stress centralities in the MEDRI model.

| **Protein/Compound** | **Clustering coefficient** | **Connectivity** | **Neighborhood connectivity** | **Stress** |
| --- | --- | --- | --- | --- |
| **AIFM1** | 0.33333333 | 3 | 15 | 1580 |
| **AKT1** | 0.24193548 | 32 | 19.6875 | 24080 |
| **AKT2** | 0.33201581 | 23 | 21.56521739 | 7974 |
| **AKT3** | 0.28571429 | 21 | 21.57142857 | 6872 |
| **ALOX12** | 0.33333333 | 3 | 13.33333333 | 84 |
| **APAF1** | 0.51515152 | 12 | 18.41666667 | 1246 |
| **APOE** | 0 | 1 | 3 | 0 |
| **ATM** | 0 | 3 | 19.33333333 | 388 |
| **BAD** | 0.16017316 | 22 | 13.59090909 | 15956 |
| **BAX** | 0.33333333 | 7 | 15 | 2428 |
| **BCL2** | 0.20261438 | 18 | 15.66666667 | 8012 |
| **BCL2L1** | 0.26470588 | 17 | 19.29411765 | 4478 |
| **BID** | 0.37426901 | 19 | 18.42105263 | 4088 |
| **BIRC2** | 0.56862745 | 18 | 19.61111111 | 1816 |
| **BIRC3** | 0.47619048 | 21 | 19.9047619 | 3094 |
| **BIRC7** | 0.66666667 | 3 | 22 | 26 |
| **BIRC8** | 0 | 1 | 21 | 0 |
| **Caffeine** | 0.4 | 6 | 13 | 1822 |
| **CAMK1** | 0 | 1 | 8 | 0 |
| **CAPN1** | 0.4 | 5 | 19 | 268 |
| **CAPN2** | 0.16666667 | 4 | 20.5 | 46 |
| **Carnitine** | 1 | 2 | 23.5 | 0 |
| **CASP10** | 0.52380952 | 21 | 20.38095238 | 2416 |
| **CASP3** | 0.19354839 | 31 | 16.93548387 | 17750 |
| **CASP6** | 0.53846154 | 13 | 19.30769231 | 1574 |
| **CASP7** | 0.38461538 | 14 | 15.5 | 1464 |
| **CASP8** | 0.44827586 | 30 | 19.83333333 | 4504 |
| **CASP9** | 0.26666667 | 21 | 15.66666667 | 6474 |
| **CAT** | 0.4 | 6 | 16.66666667 | 3452 |
| **CCNA1** | 0 | 2 | 11 | 38 |
| **CDKN1A** | 0.47619048 | 15 | 21.73333333 | 3286 |
| **CFLAR** | 0.4952381 | 21 | 20.0952381 | 2446 |
| **CHP** | 0.48888889 | 10 | 21.7 | 1120 |
| **CHP2** | 0.48888889 | 10 | 21.7 | 1120 |
| **CHUK** | 0.40740741 | 27 | 20.03703704 | 7488 |
| **CSF2RB** | 0.61818182 | 11 | 18.54545455 | 314 |
| **CYBA** | 0 | 1 | 6 | 0 |
| **CYCS** | 0.4 | 5 | 14.2 | 438 |
| **DFFA** | 0.33333333 | 3 | 15.66666667 | 30 |
| **DFFB** | 1 | 2 | 17 | 0 |
| **DLG4** | 0.3 | 5 | 6.8 | 290 |
| **DUOX1** | 0.33333333 | 3 | 14.66666667 | 1394 |
| **DUOX2** | 1 | 2 | 21.5 | 0 |
| **DYNLL1** | 0.13333333 | 6 | 13.66666667 | 1162 |
| **DYNLL2** | 0 | 2 | 9 | 72 |
| **ENDOG** | 0 | 1 | 3 | 0 |
| **EPX** | 1 | 2 | 25 | 0 |
| **FADD** | 0.46153846 | 26 | 18.53846154 | 3008 |
| **FAS** | 0.63636364 | 12 | 20.41666667 | 344 |
| **FASLG** | 0.575 | 16 | 22.125 | 1736 |
| **GPX1** | 0.66666667 | 16 | 16.25 | 14366 |
| **GPX2** | 0.75238095 | 15 | 15.6 | 7244 |
| **GRIN2D** | 1 | 2 | 10.5 | 0 |
| **GSS** | 0.85714286 | 15 | 13.73333333 | 376 |
| **GSTA1** | 0.98901099 | 14 | 14.35714286 | 2 |
| **GSTA2** | 0.98901099 | 14 | 14.35714286 | 2 |
| **GSTA3** | 0.98901099 | 14 | 14.35714286 | 2 |
| **GSTA4** | 0.98901099 | 14 | 14.35714286 | 2 |
| **GSTA5** | 0.98901099 | 14 | 14.35714286 | 2 |
| **GSTK1** | 0.98901099 | 14 | 14.35714286 | 2 |
| **GSTM1** | 0.85714286 | 15 | 13.8 | 1608 |
| **GSTM2** | 0.98901099 | 14 | 14.35714286 | 2 |
| **GSTM3** | 0.98901099 | 14 | 14.35714286 | 2 |
| **GSTM4** | 0.98901099 | 14 | 14.35714286 | 2 |
| **GSTM5** | 0.98901099 | 14 | 14.35714286 | 2 |
| **GSTT1** | 0.98901099 | 14 | 14.35714286 | 2 |
| **HSP90AB1** | 0.66666667 | 3 | 28.33333333 | 36 |
| **Hydrogen peroxide** | 0.14624506 | 23 | 13.30434783 | 21274 |
| **Hydroxyl radicals** | 0.09116809 | 27 | 9 | 30174 |
| **IKBKB** | 0.40640394 | 29 | 19.96551724 | 8446 |
| **IKBKG** | 0.42769231 | 26 | 19.88461538 | 8148 |
| **IL10** | 0 | 2 | 8 | 356 |
| **IL1A** | 0.77777778 | 9 | 11.77777778 | 62 |
| **IL1B** | 0.36666667 | 16 | 16.3125 | 3304 |
| **IL1R1** | 0.46666667 | 16 | 19.0625 | 2242 |
| **IL1RAP** | 0.63636364 | 11 | 16.27272727 | 680 |
| **IL3** | 0.5 | 5 | 15.6 | 112 |
| **IL3RA** | 0.61818182 | 11 | 18.54545455 | 314 |
| **IL8** | 0.66666667 | 3 | 19.66666667 | 2 |
| **INS** | 1 | 4 | 28.75 | 0 |
| **IRAK1** | 0.48333333 | 16 | 17.6875 | 1398 |
| **IRAK2** | 0.82222222 | 10 | 15.2 | 112 |
| **IRAK3** | 0.82222222 | 10 | 15.2 | 112 |
| **IRAK4** | 0.82222222 | 10 | 15.2 | 112 |
| **JUN** | 0.13333333 | 10 | 14.7 | 1942 |
| **Kaempferol** | 0.2 | 5 | 21.2 | 342 |
| **LDHA** | 0 | 1 | 3 | 0 |
| **LPO** | 1 | 2 | 25 | 0 |
| **MAP3K14** | 0.50988142 | 23 | 21.65217391 | 2970 |
| **Molecular oxygen** | 0.2 | 20 | 11.8 | 9402 |
| **MPO** | 0 | 1 | 7 | 0 |
| **MYB** | 0.0952381 | 7 | 12.42857143 | 3986 |
| **MYD88** | 0.57575758 | 12 | 14.58333333 | 1372 |
| **NFKB1** | 0.39855072 | 24 | 21.375 | 7040 |
| **NFKBIA** | 0.31521739 | 24 | 20.83333333 | 9586 |
| **NGF** | 0.30833333 | 16 | 22.9375 | 7788 |
| **Nitric oxide** | 0.22222222 | 10 | 20.2 | 4830 |
| **NME5** | 0 | 1 | 2 | 0 |
| **NOS1** | 0.14285714 | 8 | 7.25 | 2494 |
| **NOS2** | 0.06666667 | 6 | 8.5 | 3156 |
| **NOS3** | 0.2 | 5 | 18 | 2004 |
| **NOX5** | 1 | 2 | 21.5 | 0 |
| **NQO1** | 0 | 1 | 26 | 0 |
| **NTRK1** | 0.57777778 | 10 | 20.3 | 494 |
| **NUDT1** | 0 | 1 | 27 | 0 |
| **PIK3CA** | 0.2989418 | 28 | 16.39285714 | 9350 |
| **PIK3CB** | 0.52857143 | 21 | 17.57142857 | 3304 |
| **PIK3CD** | 0.37254902 | 18 | 16.16666667 | 3408 |
| **PIK3CG** | 0.50952381 | 21 | 17.04761905 | 3368 |
| **PIK3R1** | 0.33064516 | 32 | 16.71875 | 10944 |
| **PIK3R2** | 0.54978355 | 22 | 17.54545455 | 3318 |
| **PIK3R3** | 0.56315789 | 20 | 17.7 | 2846 |
| **PIK3R5** | 0.52631579 | 19 | 17.21052632 | 2842 |
| **POLR2G** | 0 | 2 | 13.5 | 1500 |
| **PPP3CA** | 0.46323529 | 17 | 20.05882353 | 6556 |
| **PPP3CB** | 0.55128205 | 13 | 19.84615385 | 1342 |
| **PPP3CC** | 0.62637363 | 14 | 20.57142857 | 3522 |
| **PPP3R1** | 0.53846154 | 14 | 20.92857143 | 3754 |
| **PPP3R2** | 0.48888889 | 10 | 21.7 | 1120 |
| **PRDX2** | 1 | 2 | 25 | 0 |
| **PRDX5** | 0 | 1 | 27 | 0 |
| **PRDX6** | 0 | 1 | 6 | 0 |
| **PRKACA** | 0.33333333 | 9 | 14.55555556 | 2178 |
| **PRKACB** | 0.66666667 | 4 | 13.5 | 126 |
| **PRKACG** | 0.46428571 | 8 | 14 | 2040 |
| **PRKAR1A** | 0.66666667 | 7 | 8.71428571 | 30 |
| **PRKAR1B** | 0.66666667 | 7 | 8.71428571 | 30 |
| **PRKAR2A** | 0.8 | 6 | 8.83333333 | 6 |
| **PRKAR2B** | 1 | 5 | 10 | 0 |
| **PRKCA** | 0.23333333 | 16 | 15.6875 | 7328 |
| **PRKX** | 0.5 | 4 | 12.75 | 224 |
| **PRNP** | 0 | 3 | 7.33333333 | 1892 |
| **Quercetin** | 0.20952381 | 15 | 19.73333333 | 5870 |
| **RELA** | 0.36666667 | 25 | 20.96 | 10614 |
| **RIPK1** | 0.62337662 | 22 | 20.22727273 | 2400 |
| **RNF7** | 0 | 3 | 15 | 104 |
| **SIRT2** | 0 | 1 | 3 | 0 |
| **SOD1** | 0.18681319 | 14 | 13.28571429 | 7168 |
| **Taurine** | 1 | 2 | 23.5 | 0 |
| **Theobromine** | 1 | 4 | 19 | 0 |
| **Theophylline** | 0 | 1 | 6 | 0 |
| **TNF** | 1 | 5 | 19.8 | 0 |
| **TNFRSF10A** | 0.79487179 | 13 | 22 | 548 |
| **TNFRSF10B** | 0.70588235 | 17 | 20.70588235 | 1082 |
| **TNFRSF10C** | 0.33333333 | 3 | 21.66666667 | 132 |
| **TNFRSF10D** | 0.86666667 | 6 | 19.33333333 | 132 |
| **TNFRSF1A** | 0.51778656 | 23 | 20.52173913 | 5998 |
| **TNFSF10** | 0.73076923 | 13 | 20.23076923 | 316 |
| **TP53** | 0.14461538 | 26 | 13.69230769 | 18568 |
| **TPO** | 0 | 1 | 3 | 0 |
| **TRADD** | 0.53679654 | 22 | 19.77272727 | 2960 |
| **TRAF2** | 0.71428571 | 14 | 21.85714286 | 804 |
| **TTN** | 0 | 1 | 14 | 0 |
| **VEGFA** | 0.53333333 | 6 | 24.66666667 | 308 |
| **Vitamin B12** | 0 | 1 | 27 | 0 |
| **Vitamin B2** | 0 | 1 | 27 | 0 |
| **Vitamin B3** | 0 | 3 | 9.66666667 | 4186 |
| **Vitamin B5** | 0 | 1 | 27 | 0 |
| **Vitamin B6** | 1 | 3 | 23.33333333 | 0 |
| **XIAP** | 0.46153846 | 13 | 18 | 3358 |
